# Balancing Efficiency, Stability, Scalability, and Sustainability in Hybrid Perovskite Solar Cells: A Comprehensive Review

**DOI:** 10.1021/acsomega.5c09561

**Published:** 2026-01-26

**Authors:** Anjali Varshney, Sunil Chauhan, O. Raymond Herrera, Subhash Sharma

**Affiliations:** † Department of Physics & Environmental Sciences, Sharda School of Engineering & Science, 193167 Sharda University, Plot No. 32-34, Knowledge Park III, Greater Noida, Uttar Pradesh 201310, India; ‡ Centre for Solar Cell and Renewable Energy, Department of Physics & Environmental Sciences, Sharda School of Engineering & Science, Sharda University, Plot No. 32-34, Knowledge Park III, Greater Noida, Uttar Pradesh 201310, India; § Centro de Nanociencias y Nanotecnología, 87793 Universidad Nacional Autónoma de México, Km 107 Carretera Tijuana-Ensenada, AP 14, Ensenada, B.C. 22800, México; ∥ SECIHTI - IxM - Centro de Nanociencias y Nanotecnologí́a, Universidad Nacional Autónoma de México, Km 107 Carretera Tijuana-Ensenada, AP 14, Ensenada 22800, B.C., México

## Abstract

Hybrid perovskite solar cells (PSCs) have rapidly advanced, achieving power conversion efficiencies above 27%, yet their widespread commercialization remains constrained by intrinsic and extrinsic stability issues. Recent developments in device engineering, module integration, and material composition tuning have improved through defect passivation, charge transport, moisture resistance, and enhanced durability. Apart from these, scalable fabrication techniques such as blade coating, spray coating, inkjet printing, chemical vapor deposition, and screen printing are discussed in this review, along with how these fabrication techniques impact efficiency, stability, scalability, and sustainability. Efficiency, stability, scalability, and sustainability are the four key pillars driving the promising development of PSCs. This review explains each key pillar in detail and also highlights how crucial it is to understand the physics behind each process and material interaction to achieve balanced advancement across these dimensions. It explains how these factors work together to determine the practical application of stable, high-performance, and economically feasible perovskite solar cells. This review integrates concepts from materials chemistry, device physics, and process optimization.

## Graphical abstract

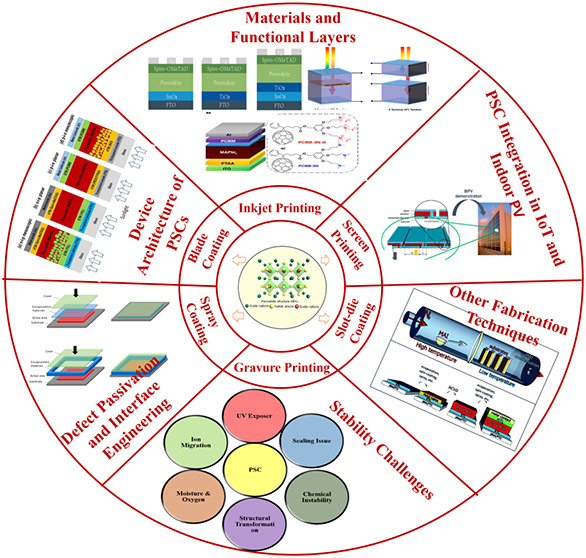

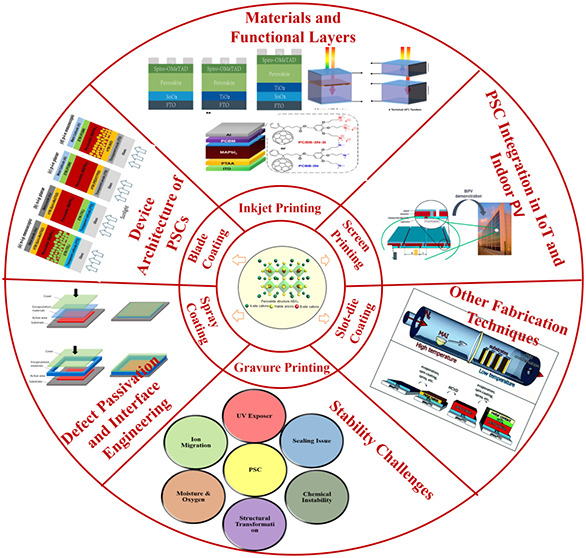

## 1 Introduction

Electricity consumption is part of contemporary life. Global energy output has not kept pace with the ongoing increase in electricity demand. Fossil fuels, including coal, oil, and gas, are now utilized as practical ways to produce power. All things considered, these fossil fuels are classified as nonrenewable energy sources, which means that eventually their supplies will run out. Additionally, a significant quantity of carbon is released into the home environment during the energy transfer process from these sources, increasing the risk to human survival. As a result, switching from fossil fuel-based energy to green, clean, and renewable energy sources is urgent and highly desired.^1^ Adopting environmentally friendly technologies in conjunction with renewable energy sources is unquestionably essential for the welfare of the globe. However, for numerous usual and renewable energy sources, including solar, wind, biomass, hydro, etc., the progress of various technologies related to energy conversion and supervision is happening at a rapid rate. Nonetheless, photovoltaic solar (PV) is seen to be the most promising and successful renewable energy available.

In particular, the planet’s most abundant renewable energy source is solar energy. According to one approximation, the sun releases 3.8 × 10²³ kW of power, while Earth recieves 1.74 × 10^14^ kW. The existing global population can use all of the solar energy the Earth receives daily in 27 years.^2^ This fact suggests that solar energy has broad applications, ranging from tiny (at the home level) to huge level (at the industrial scale). Currently, solar energy accounted for 1.9%^3^ of the 26615 TWh of global power supply in 2019, with renewable energy sources producing 26% of the total. Without the need for an interconnection, PV solar cells may directly transform light energy into electrical power. Because PV solar is simpler, it consumes less energy, produces more, and is easier to manage. With a wide variety of solar cell types that are separated into many generations, the solar PV business is a broad field. Solar cell technologies are broadly categorized into three generations based on their material composition and manufacturing approaches. First-generation solar cells primarily include wafer-based crystalline silicon devices, such as monocrystalline (c-Si) and polycrystalline (p-Si) solar cells, which dominate the current photovoltaic market due to their high efficiency and reliability. Second-generation thin-film solar cells utilize semiconductor layers applied to cost-effective substrates, commonly comprising amorphous silicon (a-Si), cadmium telluride (CdTe), and copper indium gallium selenide (CIGS) technologies. Similarly, Third-generation solar cells, including perovskite, dye-sensitized, and organic photovoltaics, emphasize economical production, adjustable band gaps, and multifunctional characteristics to exceed the performance and scalability constraints of traditional silicon and thin-film technologies. This classification structure, developed through many papers, establishes a basis for presenting perovskite solar cells (PSCs) as viable contenders in next-generation photovoltaics.^4,5^ However, the production and design of solar cells, along with their durability, reliability, and cost, remain significant obstacles.

The scientific community has focused more on PSCs than on any other type of third-generation photovoltaic technology. In 2009, the PSC was introduced as a promising photovoltaic technology for renewable energy, derived from DSSC technology. The perovskite, an active light-harvesting layer made of an organic–inorganic mixed molecule with an ABX_3_ crystal structure, is a crucial component of PSCs. After that, several artificial perovskites were produced and examined.^6,7^ The perovskite supergroup has been found to contain about 40 naturally occurring minerals in the form of hydroxides, oxides, halides, arsenides, and intermetallic complexes.^8^ The perovskite system’s categorization is shown in Figure 1A. As shown in Figure 1B,^9^ perovskites are incredibly adaptable materials that have been the upcoming star in the field of optoelectronics within the past ten years. Another PSCs Hybrid halide-perovskites, broadly classified into alkali metallic and organometallic halide types, have revolutionized solar cell research due to their superior optoelectronic characteristics. First synthesized by Dieter Weber and later structurally characterized by Mitzi and colleagues, as ABX_3_ perovskite here, X represents an anion, whereas A and B represent two cations. The general formula of perovskites is ABX_3_, where “A” represents a monovalent cation (such as Cs^+^, MA^+^, or FA^+^), “B” is a divalent metal cation (typically Pb^2+^ or Sn^2+^), and “X” is a halide anion (Cl^–^, Br^–^, or I^–^). The Goldschmidt tolerance factor gave structural stability and optoelectronic performance by affecting octahedral tilting and electrical coupling. These hybrid perovskites exhibit remarkably high absorption coefficients, facilitating efficient light harvesting in layers thinner than 500 nm, considerably less than conventional inorganic solar cells. Flexible hybrid perovskite solar cells (f-PSCs) are increasingly recognized for their use in wearable, foldable, and rollable technologies. With power conversion efficiencies reaching over 22% and compatibility with low-cost fabrication techniques like roll-to-roll printing, f-PSCs are promising for commercialization. Their high extinction coefficients and mechanical flexibility make them suitable for portable and integrated power sources in next-generation electronics. In conclusion, the synergy of excellent optical absorption, long charge diffusion, structural tunability, and mechanical compliance positions hybrid perovskites as front-runners in both rigid and flexible PV applications. Figure 1D provides a summary of the developments and notable successes for larger PSC devices and modules using various deposition techniques. According to the PSCs device and their module shown in Figure 1C, PSC devices or their modules may be broadly classified as lab cells, micromodules, submodules, and modules. Most recent research on PSCs, ranging from lab cells to scaled modules arranged, as there are currently no recognized standards for precisely classifying the devices and modules.

**Figure 1:**
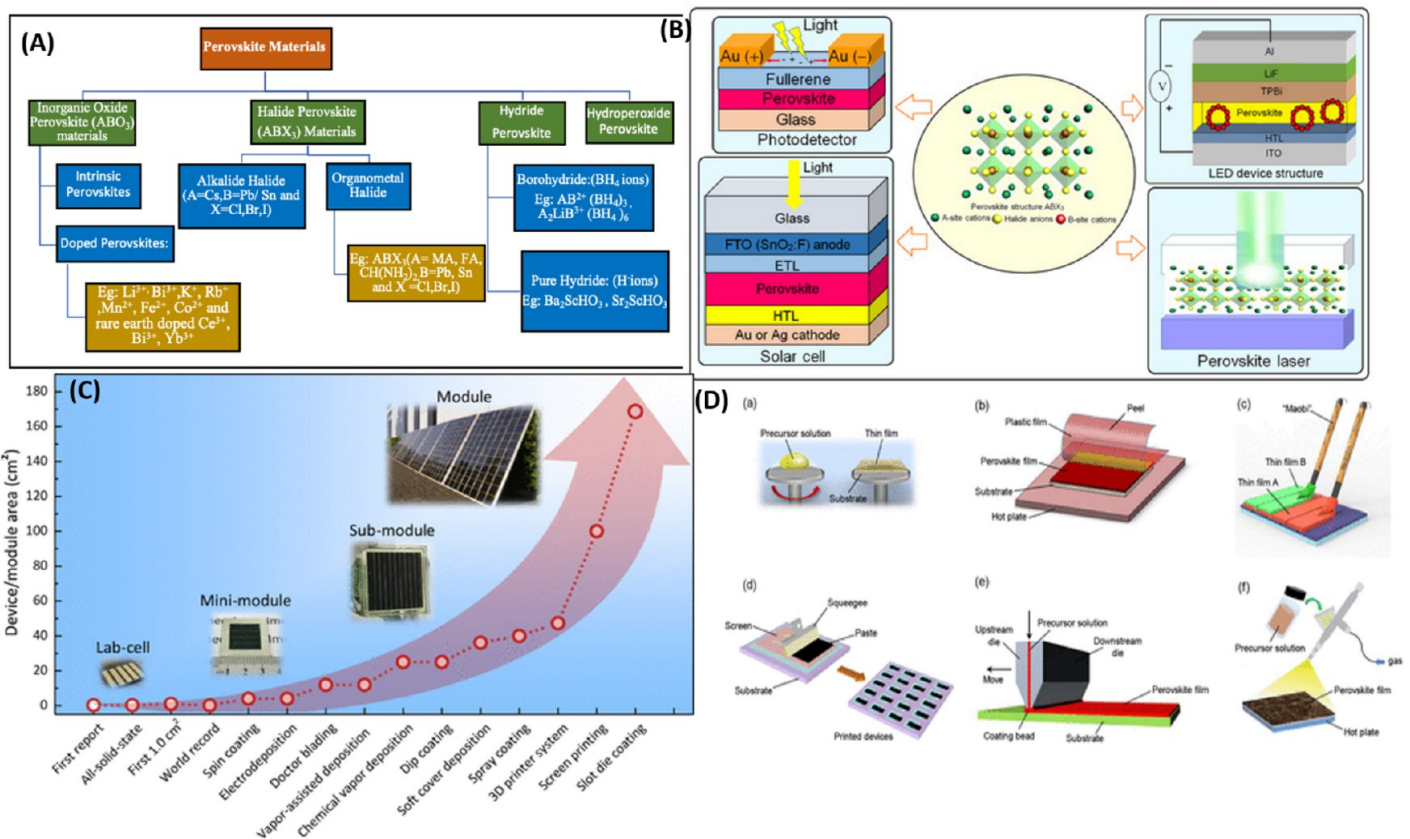
(A) Perovskite system classification.^9^ (B) Optoelectronics devices with innovative uses based on metal halide perovskite materials. Reproduced with Permission, Copyright,[Bibr ref9] 2023. (C) The development of the module of PSC devices using various deposition techniques, starting with lab cells and progressing to small modules, submodules, and finally to modules. (D) Scheme for the thin-film deposition techniques of screen printing, brush painting, slot-die coating, spin coating, soft-cover deposition, and spray coating. Reproduced with Permission, Copyright,[Bibr ref10] 2017.

Perovskites and their devices have been made using a various of technique, including spray coating, two-step deposition, dip coating, inkjet printing, chemical vapor deposition, screen printing, blade coating, and more (Figure 1D).^10–22^ Hybrid PSCs have revolutionized photovoltaic research, achieving an effective efficiency increase from the range 4% to 27% within a span of just over 10 years.^11^ Nonetheless, despite swift progress, obstacles in long-term stability, scalable production, and environmental sustainability persistently hinder their commercialization. The study’s next part included a schematic representation of the preparation methods. Specifically, each approach has demonstrated certain benefits and limitations. It should be mentioned that perovskites are already widely known to have exceptional and perfect properties for solar cell uses, including a direct bandgap, broad spectrum absorption, high defect tolerance, and charge carrier diffusion lengths ranging from micrometers.

In 2009, Kojima et al. published the first study on PSC using CH_3_NH_3_PbI_3_ perovskite.^20^ To create a PSC with a TiO_2_ scaffold perovskite absorber layer, they employed a liquid electrolyte as the hole transport layer (HTL). The PCE that was reported was 3.8%. Nevertheless, it has been shown that perovskites dissolve in these electrolytes, leading to rapid degradation in less than an hour.^21^ Kim et al. (2012) used solid-state HTL to help increase stability and PCE.^15^ Spiro-OMeTAD (2,2′,7,7′-tetrakis (N, N-dip-methoxyphenylamine)-9,9′-spirobifluorene) was utilized in place of liquid electrolyte, which is often used in organic LEDs. The device was proven to be stable for 500 h at room temperature without encapsulation, with an obtained PCE of 9.7%. Among the significant advancements noted by Lee et al. that year was an increase in PCE of up to 10.9%.^16^ They created CH_3_NH_3_ PbI_3‑x_Cl_*x*_, a mixed halide perovskite, and discovered that it was capable of carrying both electrons and holes. They also use insulating Al_2_O_3_ in place of the TiO_2_ scaffold layer. Additionally, compared to CH_3_NH_3_PbI_3_ perovskite, the device exhibits superior stability and charge transport characteristics. Recently, hot carrier solar cells (HCSCs) based on perovskite absorbers have attracted growing attention for surpassing the Shockley–Queisser limit by collecting carriers before thermalization. Researchers utilizing a methylthio-substituted phthalocyanine-based (SMePc) hole transport layer achieved PCE of 24.43% under 1 sun and 27.30% under 5.9-sun illumination, demonstrating excellent thermal stability and efficient hot-hole extraction. This review seeks to address this gap by providing a comprehensive explanation of efficiency, stability, scalability, and sustainability—the trinity that collectively provides the practical and technological implementation of perovskite solar cells

## 2 Recent Advances

Hybrid perovskites are an emerging class of materials with enhanced potential for multiple applications. This kind of research on materials is still ongoing today, and the expected stable and good, as well as the lead-free, which have not yet been achieved. The tunable bandgap,^23,24^ high optical absorption coefficient, low exciton binding energy,^25,26^ and balanced charge carrier mobility with minimal defect formation^27^ play a crucial role in it. Among these, methylammonium-based lead halide perovskites have been extensively studied. Owing to these attributes, hybrid perovskites have rapidly transformed the photovoltaic field, with the PCE of PSCs soaring from the range 4% to 27% within a remarkably short time span.^28–30^ Table 1 summarizes the development of hybrid perovskite solar cells employing different HTMs, along with their corresponding power conversion efficiencies (PCEs).^28,30–35^ Apart from these, some other research also reported extraordinary PCE.

**Table 1:** Comparative List of Perovskite Composites Based on Their Efficiency and Stability from 2010 to 2025

No.	Perovskite Composition/Type	HTM/Device Structure	Deposition Method	PCE (%)	Year	Refs
1	CH_3_NH_3_PbI_3_ Quantum Dots	I/I_3_^–^ Electrolyte, TiO_2_ substrate	Solution process	6.5	2015	^28^
2	CH_3_NH_3_PbI_3_	CuI, TiO_2_/CH_3_NH_3_PbI_3_/spiro-OMeTAD	Spin coating	6.0	2015	^43^
3	CH_3_NH_3_PbI_3‑*x*_Cl_*x*_	NiO, FTO/NiO/CuSCN/CH_3_NH_3_PbI_3_-_*x*_Cl_*x*_/PCBM/Ag	Spin coating	7.3	2016	^33^
4	CH_3_NH_3_PbI_3_	CuSCN, TiO_2_/CH_3_NH_3_PbI_3_/CuSCN/Au	Solution process	12.4	2016	^34^
5	CH_3_NH_3_PbI_3‑*x*_Br_*x*_	PTAA, TiO_2_/CH_3_NH_3_PbI_3‑*x*_Br_*x*_/DMSO/PTAA/Au	Antisolvent method	16.2	2017	^31^
6	CH_3_NH_3_PbI_3_	NiOx, ITO/NiOx/CH_3_NH_3_PbI_3_/PCBM/Ag	Solution processed	16.47	2017	^35^
7	CH_3_NH_3_PbI_3‑*x*_Cl_*x*_	Spiro-OMeTAD, ITO/PEIE/TiO_2_/CH_3_NH_3_PbI_3‑*x*_Cl_*x*_/spiro-OMeTAD/Au	Spin coating	19.32	2018	^30^
8	CsPbI_2_Br (All-inorganic)	ITO/SnO_2_/MOH/CsPbI2Br/carbon	alkali hydroxide (KOH, NaOH) interfacial engineering	11.78	2019	^44^
9	Cs_2_AgBiBr_6_	ITO/SnO_2_/Cs_2_AgBiBr_6_/Au	Heterojunction	-	2018	^45^
10	(FAPbI_3_)_0.95_(MAPbBr_3_)_0.05_	ITO/SnO_2_/perovskite/spiro-OMeTAD/Au	Antisolvent spin coating	22.6	2025	^46^
11	(MA_0.85_FA_0.15_)Pb(I_0.85_Cl_0.15_)_3_	FTO/SnO_2_/AEP/Spiro-OMeTAD/Au PSCs	Two-step spin coating	28.36	2025	^47^
12	Ag_3_AuSe_2_-based hybrid perovskite solar cells	Al/FTO/SnS_2_/Ag_3_AuSe_2_/CFTS/Au	Spin coating	29.60	2025	^48^
13	MAPbI_3_/FAPbI_3_–Based	charge transporting layer (CTLs)	thermal evaporation	20.08	2024	^49^
14	biomass-derived, furan-based conjugated polymer, PBDF-DFC	Direct Integration	one-step solution processing	21.39	2025	^50^

For a monolithic tandem solar cell, which maintained over 80% of its initial efficiency at 85 °C in air over 300 h, Leijtens et al. reported raising the PCE to 19.1%.^17^ Additionally, Akin reported a PSC with a PCE of 22% that exhibits outstanding stability under constant 1.0 solar light for 2000 h.^22^ According to Zhou et al.,^36^ low humidity (RH 30%) during fabrication controls the production of perovskite crystals and raises the PCE to 19.3%. Using Cu doped NiO_*x*_ as an HTL, Chen et al.^37^ developed high-efficiency devices with a PCE of 17.41% on flexible substrates and 20.26% on rigid substrates in 2018. The inverted-structure devices demonstrated consistent performance over 1000 h in ambient circumstances with high humidity (50–65%). Alkali chloride interface modification of the NiO_*x*_ hole transport layer has also been reported to improve the ordering of the perovskite films. This, in turn, reduces interfacial recombination, suppresses ion migration, lowers defect/trap density, and boosts device stability and efficiency.^38^ According to Song et al. reports, the efficiency of the PSC may be increased by p–p^+^ homojunctions, such as NiO/Cu: NiO bilayer, which is greater than using NiO or Cu: NiO alone as HTM. Specifically, the homojunction enhances hole extraction and stimulates carrier recombination at the NiO/perovskite interface at the same time by accelerating hole transfer and blocking electron transport in the HTL. The PSC with bilayer HTM maintained 80% or so after 350 h with great functioning stability and a PCE of 18.3%.^39^ Min et al. recently created PSCs with the greatest PCE of 25.8% by reducing the interface flaws between the perovskite absorber layer and SnO_2_ ETL by interlayer modification.^40^ After 500 h of constant light exposure, the gadgets retained about 90% of their original efficiency. Their results demonstrated that reducing interface flaws might increase device stability and efficiency. Although the stability is still not up to par with those commercial solar cells, it is readily apparent that the efficiency increased from 3.8% to 25.8% in a matter of years, surpassing several well-known commercial PV technologies, such as Si, CdTe, and GaAs-based solar cells. One of the primary causes is the presence of unstable elements in perovskite materials as a result of additional weak interactions, including weak hydrogen bonds and van der Waals forces.^40^ Furthermore, the environmental factors like humidity, heat treatment, and sunlight all affect how stable perovskite technology.^41^ Bass et al.^42^ suggest that humidity may control the crystallization of perovskites. Similarly, lots of researchers have done a lot of work to enhance the PCE and make it useful in a lot of applications.

The hybrid halide perovskite structure, characterized by the general formula ABX_3_, offers a very adaptable lattice wherein A-site cation elements such as MA^+^, FA^+^, Cs^+^, B-site metal cations such as Pb^2+^, Sn^2+^, Bi^3+^, or Ag^+^, and X-site halides such as I^–^, Br^–^, Cl^–^ can be manipulated to optimize optoelectronic capabilities. Concurrently, interface engineering utilizing advanced hole transport materials (HTMs) such as spiro-OMeTAD, PTAA, NiO_*x*_, and CuSCN has reduced interfacial recombination, while scalable deposition techniques, including blade coating, slot-die printing, and vacuum-assisted crystallization, have advanced PSCs toward industrial viability. Overall, the experimental developments summarized in the Table 1 collectively demonstrate the rapid maturity of perovskite technology.

The future direction involves the integration of nontoxic materials, defect-passivating interfaces, and scalable manufacturing techniques to ensure long-term operational stability while preserving high efficiency, thus achieving the four-fold objective of balancing efficiency, stability, scalability, and sustainability.

## 3 Four Pillars in Hybrid Perovskite Solar Cells

### 3.1 Overview of Key Challenges and Strategic Direction

Perovskite materials are known to be unstable, and it has been confirmed that many degradations take place concurrently at different interfaces at different levels of the device. This degradation can be directly caused by UV rays, high temperatures, moisture, and exposure to the outside air. As with other materials made of organic components, the reasons for the stability problem may thus be identified as inherent stability, moisture intrusion, oxygen ingress, and illumination-accelerated degradation.^51,52^ The structural and chemical stability of devices under a variety of photovoltaic operating circumstances, including the presence of impurities like water and oxygen that were added during manufacture, is referred to as intrinsic stability. Extrinsic stability addresses the shortcomings of moisture-blocking and sealing layers. Degradation processes are triggered or accelerated under normal circumstances. Figure 2(A-D) schematically depicts the instability problems. Consequently, the previously described factors may influence the material’s organic layer disintegration, photodegradation, top electrode instability, and crystallization instability. Because of the inherent deterioration caused by heat stress or light, a variety of degradations can start even in the absence of oxygen or water.^53^ The instability of the used charged transport material and the intrinsic instability of the perovskite material are both to blame for the stability shortcomings.^54^ These challenges remain in the PSCs fabrication process, which is discussed in the next part. Still not yet able to manufacture high-efficiency PSCs on a large scale or in atmospheric conditions. Limitation taken because the synthesis operations must be finished in a glovebox or other controlled, inert environment, since perovskites are extremely sensitive to moisture and oxygen.^55^

**Figure 2:**
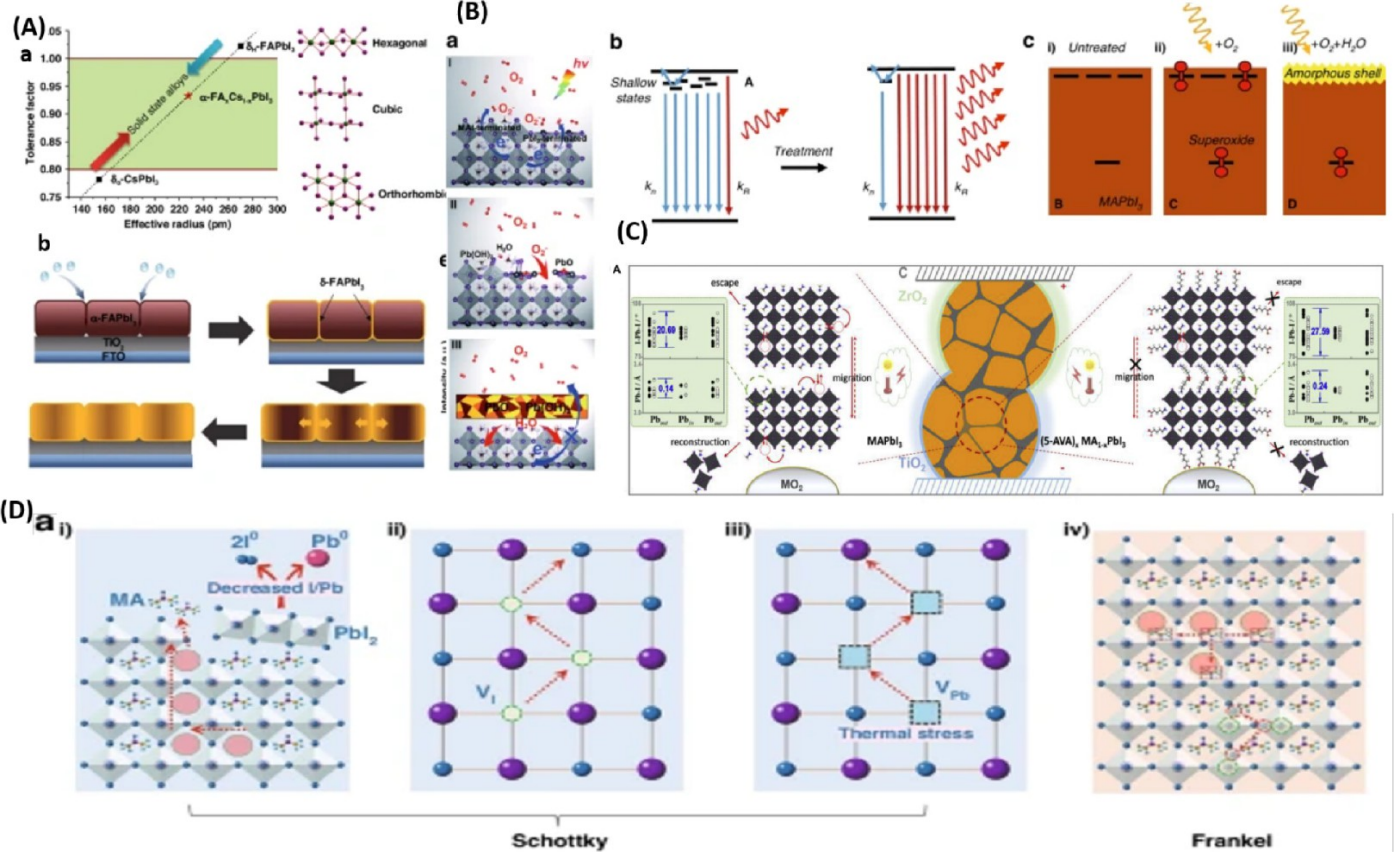
(A) a- The tolerance factor and the perovskite crystal structure are correlated. Reproduced with permission Copyright,^61^ b- Schematic showing the many stages of the deterioration process, with permission. Reproduced with permission Copyright,[Bibr ref62] 2019. (B) a- A diagram showing the MAPbI_3_ (001) surface’s photo-oxidative degradation pathway. Reproduced with permission, Copyright,[Bibr ref63] 2017. b- Diagram showing how the existence of shallow surface states causes nonradiative recombination (kn) supremacy to change to radiative-dominant recombination (kR) once these states are eliminated by treatment. Film. c- (i) of untreated MAPbI_3_ contains nonradiative trap states that passivate when MAPbI_3_ is subjected to (ii) oxygen and light, and (iii) oxygen, light, and water. Reproduced with permission, Copyright,[Bibr ref64] 2020. (C) Diagrams showing the MAPbI_3_ and (5-AVA)_*x*_MA_1–*x*_PbI_3_ structural arrangements in triple-mesoscopic layers, as well as the processes of material breakdown and ionic migration brought on by the interaction of light, heat, and electrical bias. Details of the Pb–I bond lengths and I–Pb–I bond angles in MA ± terminated slabs (left) and 5-AVA ± terminated slabs (right) are shown in the green insets. Reproduced with permission, Copyright,[Bibr ref65] 2023. (D) A diagram showing the types of defects and ion migration in MAPbI_3_ after it has been heated to 85 °C. Schottky defects include migration of a (i) MA^+^, (ii) I^–^, (iii) Pb^2+^, and (iv) Frankel defects, as well as vacancy defects. Reproduced with permission, Copyright,[Bibr ref66] 2025.

Furthermore, significant perovskite degradation is seen when the constructed devices are tested in ambient circumstances. The usage of PSCs outdoors may be limited due to the unintended reduction in efficiency brought on by PSC degradation.^56^ The effects of air and moisture on the potency and efficacy of PSCs are well documented.^57^ Additionally, PSCs deteriorate in the presence of light. When air and UV light are present, CH_3_NH_3_I generates a brown color, which Ponseca et al. ascribed to the presence of I_2_.^57^ The breakdown of perovskite materials in the presence of air and UV light may be the source of this I_2_. The thermal stability of PSCs is a major challenge in addition to stability issues with oxygen, moisture, and UV radiation.^58^ Exposure to high temperatures can cause the perovskite layer to degrade. It is important to remember that the solar modules will be exposed to temperatures of up to 100 °C when in use. Djurišić et al. investigated the effects of elevated temperatures on CH_3_NH_3_PBI_3‑x_Cl_*x*_ and CH_3_NH_3_PBI_3_.^59^ XPS was used to explain the breakdown of these films since the technique is more appropriate for examining the chemical components of materials, independent of their crystallinity. The film was heated to 100 °C in an ultrahigh vacuum chamber without any air or moisture to assess the impact of temperature alone on the breakdown of the film. The films were characterized using the N/Pb and I/Pb ratio that XPS had found. As may be shown elsewhere, the decrease in these ratios shows how the perovskite material changes into lead iodide.^59^ According to this elemental analysis, perovskites cannot tolerate temperatures higher than 100 °C. Furthermore, it was noted that the CH_3_NH_3_PbI may break down in an inert environment during annealing at 85 °C.^60^ According to reports, the XA cation could be in charge of the thermal stability, which might be improved by changing the cation element. According to other studies, PSCs, which are unencapsulated cells-degraded over several hundred hours when showing to air with humidity levels higher than 50%.^61^ Gradually, the phase transition moves from the grain borders inward toward the grain interior. Figure 2A­(a,b) provides a visual representation of these dynamic processes.^62^

In other words, hybrid PSCs have revolutionized the photovoltaic landscape, achieving PCEs surpassing 26% within a decade. However, the translation of laboratory-scale breakthroughs into commercial devices is constrained by four interrelated challenges—efficiency, stability, scalability, and sustainability. The properties of perovskites with tunable bandgap, elevated absorption coefficients, and extended carrier diffusion lengths, provide good potential for PSCs performance. however, their chemical and structural stability to moisture and heat, and dependence on toxic lead compounds, present significant obstacles. To resolve some of the challenges toward PSCs, researchers have proposed various solutions: interface passivation and compositional engineering to improve efficiency, encapsulation and additive techniques for prolonged stability, scalable deposition methods for consistency, and lead-free or recyclable strategies to guarantee sustainability. The subsequent subsections analyze each pillar, including the fundamental scientific challenges and the associated technology solutions to address them.

#### 3.1.1 Efficiency: Overcoming Photophysical and Interfacial Challenges

The PCE of hybrid PSCs depends mainly on how well they can create, move, and collect the charge carriers generated by light. However, many physical and interface-related problems can slow down or block this process, reducing the efficiency and stability of the solar cells. A significant problem stems from nonradiative recombination, which is caused by deep trap states and dangling bonds at grain boundaries, interfaces, and surface defects. Potential barriers frequently arise at the interfaces as a result of the energy level mismatch between the perovskite absorber and the nearby charge transport layers, such as the HTL and ETL. These obstacles hinder the transfer of charges and encourage the buildup of carriers. Additionally, poor band alignment and restricted hole mobility in these layers may degrade device performance. The overall efficiency of perovskite solar cells is further limited by optical and electrical losses, which are caused by parasitic absorption in transport layers, inadequate light management, and ineffective charge extraction. Numerous interfacial engineering techniques have been created to overcome these issues. For example, in p-i-n-type devices, copassivation using phenylethylammonium iodide and 4-methoxyphenylphosphonic acid efficiently suppresses nonradiative voltage losses to about 59 mV and minimizes interfacial trap states, leading to a constant power conversion efficiency of 25.53%. Additionally, it has been demonstrated that self-assembled monolayers with heteroatoms like oxygen or sulfur can improve surface passivation, decrease interfacial recombination, and modify the work function, leading to power conversion efficiencies of over 24–26% in inverted topologies. Furthermore, 1,6-hexylenediphosphonic acid surface modification increases carrier lifetimes and suppresses nonradiative losses, which enhances device performance. These results highlight how crucial it is to combine compositional engineering techniques with interfacial optimization to achieve the theoretical performance limits of perovskite solar cells.^67–71^ The following section goes into additional detail about ongoing developments via material Innovation, compositional change, interfacial passivation, and device architectural improvement.

##### 3.1.1.1 Material Innovations

Organic–inorganic hybrid perovskite solar cells (PSCs) are a revolutionary class of photovoltaic materials due to their high-power conversion efficiencies (PCEs), affordability, and solution-processing capabilities. Beneficial optical and electrical characteristics, such as a high absorption coefficient, prolonged carrier diffusion length, low defect density, and tunable bandgap, were demonstrated by the integration of organic and inorganic materials.^73,74^ PCE of PSCs has risen quickly from 3.8% to about 28%, outperforming several traditional thin-film photovoltaic technologies.^75^ The potential of perovskite absorbers for upcoming cost-effective and highly efficient solar solutions is highlighted by this advancement. Perovskite materials can be processed in solution at lower temperatures using scalable deposition techniques like blade coating, slot-die coating, or vapor-assisted growth, in contrast to silicon photovoltaics, which require high-purity silicon processing at temperatures above 1000 °C and intricate fabrication methods like texturing and antireflective coating application.^76,77^

Large-area module production still faces stability and efficiency issues in spite of these improvements. Using cation or anion substitution, such as the combination of FA^+^/MA^+^ or the partial substitution of Sn^2+^ or Bi^3+^ for Pb^2+^, this overcomes certain constraints of compositional engineering. This demonstrates the ability to modify bandgap tunability and improve structural stability. At the same time, trap-assisted recombination has been greatly reduced and moisture resistance has been improved through defect passivation using molecular and ionic additives such as zwitterions, K^+^, or Cs^+^.^78^ The stage of PSC development links material chemistry to enhanced device performance and operational stability, facilitating a smooth transition to the advanced compositional engineering discussed in the subsequent section.

##### 3.1.1.2 Compositional Engineering

Material compositional engineering remains the cornerstone of performance enhancement in PSCs. Compositional and defect passivation strategies directly provide stability and electronic properties of the film, laying the foundation for efficient and robust device architectures. A potential approach to enhancing device efficiency, environmental stability, and scalability is compositional engineering of 2*D*/3D hybrid PSCs. Despite their efficiency, traditional 3D perovskites like MAPbI_3_ and FA-based systems have poor moisture and temperature stability. Researchers started adding big organic spacer cations like BA^+^, PEA^+^ to create quasi-2D or 2D/3D layered perovskite heterostructures to solve these problems. These low-dimensional architectures allow for high charge carrier transit via the embedded 3D phases while providing improved stability through hydrophobic surface layers and inhibited ion migration. By adjusting the “n” value, compositional control has made it possible to precisely balance stability and efficiency.

For instance, Mohanty et al. used BA_2_MA_4_Pb_5_I_16_ (n = 5) to establish a graded 2*D*/3D perovskite interface in 2021, obtaining a certified PCE of 23.3% with enhanced moisture resistance.^79^ Similar to this, in 2025 Patil et al. tailored the orientation and crystallinity of quasi-2D films using mixed spacer cations such as PEA^+^ and iso-BA^+^. This resulted in efficiencies of up to 24.1% with little hysteresis and 1,000-h operating stability under 85 °C.^49^ In 2023, PSCs with open-circuit voltages reter than the 1.2 V were made possible by the successful surface passivation and decreased defect densities demonstrated by the combination of 2D perovskites with Ruddlesden–Popper phases (e.g., (PEA)_2_(MA)_4_Pb_5_I_16_).^80^ To further improve stability and grain boundary passivation, compositional additives such as thiocyanate, guanidinium (GA^+^), and trace quantities of Br^–^ or Cl^–^ have been added.^72,85^ The creation of semitransparent and wearable PSCs is made possible by the synergy of 2*D*/3D stacking, which also provides compatibility with flexible substrates. According to Yuan et al.,^81^ scalable blade-coating of 2*D*/3D perovskite films utilizing green solvents such as dimethyl sulfoxide (DMSO)/IPA combinations allowed for the low-cost, roll-to-roll manufacture of solar modules with PCEs exceeding 21% and exceptional mechanical durability. All things considered, compositional engineering of 2*D*/3D hybrid structures has closed the gap between long-term stability and efficiency, signaling a significant shift in the viability of perovskite photovoltaics for commercial use. When compared to hybrid perovskites, inorganic perovskites with the general formula CsBX_3_—where B is for Pb or Sn and X is a halide—have drawn more interest due to their superior thermal stability.^82–84^ These materials are appropriate for tandem solar cells that require high open-circuit voltages because of their adjustable bandgaps, which range from 1.73 to 2.20 eV. Solar cells are a relatively new application for cesium-based perovskites, despite their discovery in the late 1800s. For example, early CsSnI_3_ cells had extremely low efficiencies of roughly 0.8%, but thanks to changes like Ge doping, efficiency increased to 7.11% by 2019. Later, more stable Pb-based perovskites like CsPbI_3_ and CsPbBr_3_ were created to address the issue of Sn^2+^ oxidation. CsPbI_3_ has advanced significantly among them, achieving power conversion efficiencies of up to 21%.^86–88^

The use of costly metal electrodes like gold and silver and hole transport layers like Spiro-OMeTAD or PTAA, in spite of these advancements, increases production costs and may lead to device degradation because metal ions can enter the perovskite layer.^89,90^ In order to get over these problems, scientists are looking into less expensive options such as carbon and aluminum electrodes as well as affordable hole transport layers like NiO and CuSCN.^91–94^ Due to their abundance, chemical stability, water resistance, and ability to stop ion migration from the perovskite, carbon electrodes hold great promise for long-lasting and robust solar cell applications.^89,93,95,96^ The first carbon-based CsPbBr_3_-PSC was reported in 2016 with 5% PCE,^97^ and recent devices have achieved certified efficiencies of up to 14%.^98,99^ These advances suggest carbon-based inorganic PSCs (C-IPSCs) could play a key role in scalable and low-cost photovoltaic technologies. PSC materials utilized in these cells interact with oxygen and moisture in the surrounding environment.^100,101^ As a result, the research community is concentrating on nontoxic, stable, and effective materials. Thus, the different substitutes for methylammonium (MA) at site A are formamidine (FA), cesium (Cs), or a combination of the three; at site B, the substitutes are bismuth (Bi), tin (Sn), silver (Ag), germanium (Ge), antimony (Sb), etc. Cesium is a viable candidate for the A position since CsPbI_3_ has an impressive efficiency of around 19% and CsPbI_2_Br has an efficiency of about 16.7%, even though both PSCs contain toxic Lead.^102,103^ PSCs are lead-free and ecologically benign when Pb is substituted with tin. The most often reported PSCs devoid of lead in the literature are CsSnI_3_, CsSnI_2_Br, CsSnIBr_2_, and CsSnBr_3_. However, the fast oxidation of Sn^2+^ to Sn^4+^ hinders these tin-based PSCs. When SnF_2_ was introduced to CsSnI_3_, CsSnI_2_Br, CsSnIBr_2_, and CsSnBr_3_, Sabba et al. observed efficiencies of 1.66%, 1.67%, 1.56%, and 0.95%, respectively, in 2015.^104^ Adding hypophosphorus acid to CsSnIBr_2_PSC decreased Sn vacancies and prevented the rapid oxidation of Sn^2+^ to Sn^4+^ by around 3%, according to a 2016 study by Li et al.^105^ By improving defect and charge transfer port layers in CsSnIBr_2_-based PSCs using the SCAPS-1D simulation program, Raouit et al. recently obtained 20.32% efficiency.^106^ Similarly, Elango Group used a comparable modeling technique to optimize thickness, defect density, bandgap, and electron affinity, demonstrating 28% efficiency.^107^

Similarly, another group reported about 17% efficiency in all inorganic CsSnBr_3_-based PSC using a comparable simulation method.^108^ Pb-free PSC based on CsGeI_3_ has advantageous optoelectronic features and reaches 0.11% efficiency, as shown by Krishnamoorthy et al.^108^ In 2018, Chen reported a solvothermal process efficiency of 4.92%.^109^ The A_2_B^+^B^3+^X_6_ elpasolite double perovskite (DP) structure may be created by substituting one monovalent B+ and one trivalent B^3+^ ion for the Pb^2+^ ion.^110^ Despite being more stable than PSCs, DPs are less effective. Wu et al.^111^ produced a Cs_2_AgBiBr_6_-based DP with a 1.44% efficiency. In contrast, the Yang group extends solar spectrum absorption by adding an extra N719 dye interlayer, improving performance to 2.84%.^112^ Zhang et al. recently increased the efficiency to 6.37% by using hydrogenated Cs_2_AgBiBr_6_ DPs.^113^ By employing DFT studies to scan another DP, Cs_2_TiI_6_, the Zhao group demonstrated outstanding performance, achieving 22.70% efficacy in single junction and 26.87% efficiency in tandem uses.^114^ According to the literature review, compositional engineering successfully modifies the bandgap, inherent stability, and crystal structure of perovskites, uncoordinated ions, grain-boundary traps, and interfacial defect states continue to be the source of several significant performance losses. Therefore, targeted defect passivation techniques become a crucial supplementary strategy to further inhibit nonradiative recombination and achieve long-term operational stability.

##### 3.1.1.3 Defect Passivation

The performance loss in PSCs compared to their theoretical maximum is mainly due to lower open-circuit voltage (*V*_OC_) and fill factor (F_F_), which are affected by recombination losses caused by defects at interfaces or within the bulk. These defects act as Shockley–Read–Hall (SRH) recombination centers.^115^ Since perovskite materials easily form such defects due to low formation energy,^116^ it is essential to passivate them to improve performance. Many of these defects arise from uncoordinated Pb^2+^ or halide ions (I^–^, Br^–^), particularly on the surface and grain boundaries.^117–119^ Additive structure using Lewis acid and base molecules is one of the most promising strategies for defect passivation.^120–122^ Lewis acids accept electron pairs, effectively neutralize uncoordinated halide ions and antisite defects. Common Lewis acid additives include cationic dopants, fluorinated aromatics, and fullerene derivatives.^123–127^ For instance, Rb^+^ ions act as efficient cationic Lewis acids; doping with RbI helps suppress the unwanted δ-phase in formamidinium FA-based perovskite, improving charge carrier lifetimes and reducing hysteresis (Figure 3a).^128^ Fluorinated aromatic molecules like iodo-penta-fluorobenzene (IPFB) use strong halogen bonds to fix defects where iodine replaces lead (Figure 3b). Tetra-fluoro-phthalo-nitrile (TFPN) binds uncoordinated lead ions and shifts energy levels to improve hole movement (Figure 3c).^129,130^ Fullerene derivatives such as PCBM passivate iodide-rich traps at grain boundaries, helping electron flow and reducing hysteresis (Figure 3d).^131,132^ The acidity (p*K*_a_) of additives matters: cyclo-hexyl-ammonium chloride (CYCl) with high p*K*_a_ leads to better defect passivation and efficiency than anilinium chloride (ANCl), which releases free iodide ions causing defects (Figure 3e,f). Lewis bases containing nitrogen, oxygen, or sulfur donate electrons to neutralize positively charged defects.^133–135^ Nitrogen-based additives, like ethylenediamine chloride (EDACl_2_), lower trap states and boost charge transport. However, basic Lewis bases can also harm the perovskite structure by deprotonating cations, so they must be carefully chosen. Oxygen-based bases like 1,2,4,5-benzenetetracarboxylic acid (BTCA) reduce ion movement and trap density, improving voltage (*V*_OC_) to 1.076 V. Molecules such as 4-(dimethylamino)­benzylamine (DLBA) with strong dipoles passivate lead defects well (Figure 3g). Sulfur-based bases like 2-mercaptopyridine (2-MP) form strong bonds with lead ions, protecting films from moisture and increasing efficiency to 20.28% PCE and 1.18 V of *V*_OC_ (Figure 3h,i). Overall, using both Lewis acid and base additives together effectively cuts defects, lowers recombination, and improves PSC efficiency and lifespan. Proper design and understanding of their interaction with perovskite are crucial for success.^129–141^ Even defect passivation mitigates trap-assisted losses, in literature researchers reported that the overall device performance is equally governed by the underlying device configuration. Therefore, the next section discusses how different device architectures further influence charge extraction, stability, and efficiency

**Figure 3:**
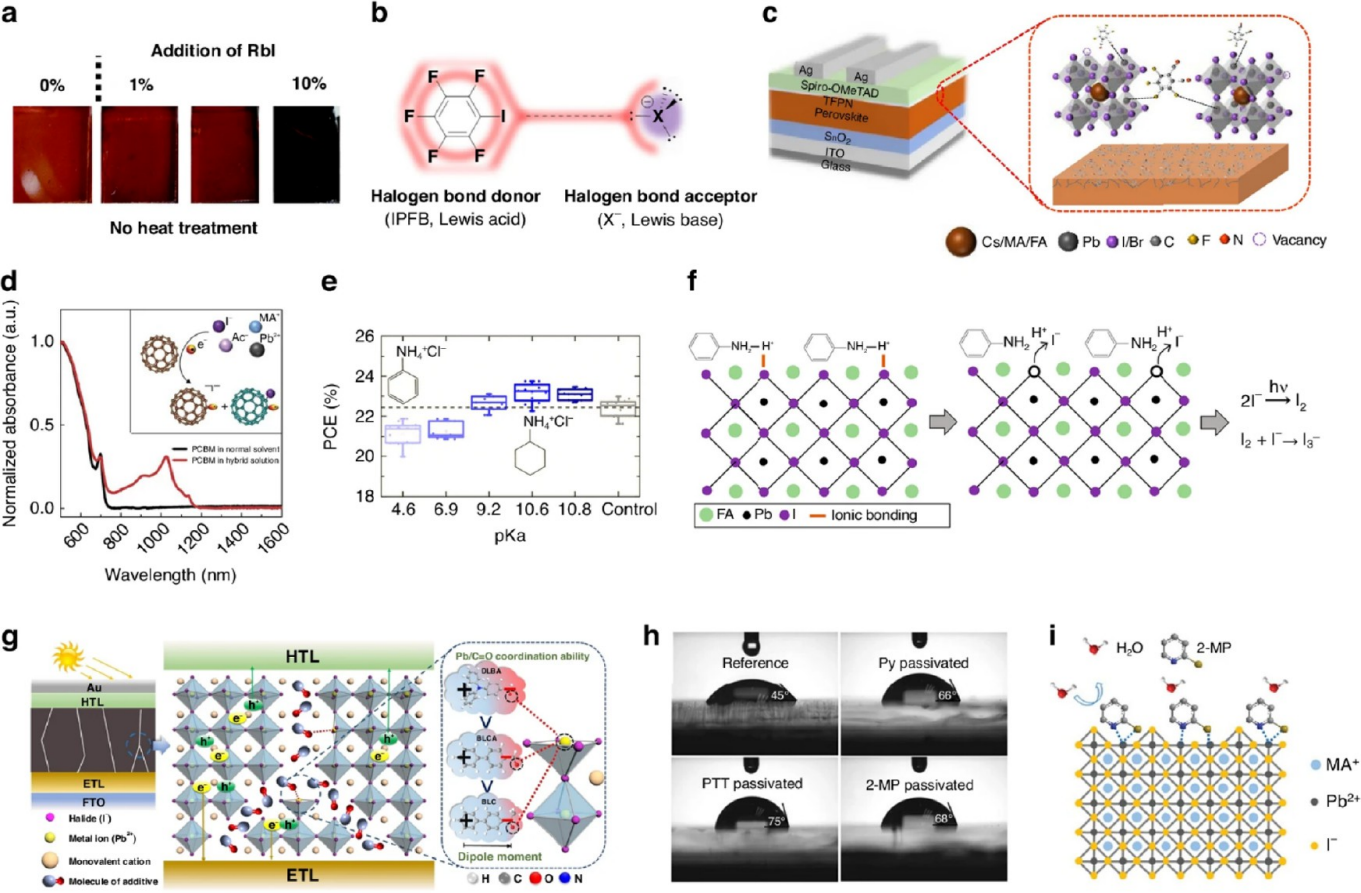
(a) Charge carrier dynamics at room temperature of perovskite films containing Rb^+^ with enhanced lifetimes. Reproduced with permission, Copyright,[Bibr ref128] 2018. (b) Diagram showing the noncovalent defect passivation in the halogen bond interaction between Iodopentafluorobenzene (IPFB) and halogen anion. Reproduced with permission, Copyright,[Bibr ref117] 2014. (c) Tetrafluorophthalonitrile’s (TFPN) molecular structure and how it interacts with uncoordinated Pb^2+^ to passivate it. Reproduced with permission, Copyright,[Bibr ref130] 2021. (d) The hybrid solution’s UV absorption spectra demonstrate how [6,6]-phenyl-C_61_-butyric acid methyl ester (PCBM) and perovskite ions interact. Iodide interaction forms PCBM radical anion and PCBM–halide radicals, as seen in the inset. Reproduced with permission, Copyright,[Bibr ref132] 2015. (e) The relationship between p*K*_a_ values and PCE for various additives shows how additive acidity affects device performance. (f) A schematic representation of the chemical interactions that occur between ammonium chloride (ANCl) and perovskite surfaces, demonstrating how iodide defects can result from low-p*K*_a_ additions. Reproduced with permission, Copyright,[Bibr ref137] 2021. (g) Molecular polarity influence is highlighted in the action mechanism diagram of additive dipole effects from DLBA, BLCA, and BLC on perovskite films. Reproduced with permission, Copyright,[Bibr ref140] 2023. (h) Enhanced moisture stability is demonstrated by a visual comparison of unpassivated and 2-MP passivated perovskite films before and after exposure to high humidity. (i) Mechanistic schematic of 2-MP passivation, where the energy barrier against moisture attack is increased by high Pb^2+^ binding. Reproduced with permission, Copyright,[Bibr ref141] 2019.

##### 3.1.1.4 Device Architecture

The performance and efficiency of hybrid PSCs depend on their device architecture, which controls movement and improves energy conversion efficiency of charge carriers. Optimizing the device setup works alongside material improvements by addressing charge transport, recombination, and interface wear. PSCs have several layers, each with a specific job: the perovskite layer absorbs sunlight and creates electron–hole pairs. Charge transport layers include an ETL that moves electrons to the cathode, and an HTL that moves holes to the anode. The device is held between electrodes, usually a transparent conductive oxide like ITO or FTO in front, and a metal contact such as gold, silver, or carbon at the back.^142,143^ Depending on the order and choice of layers, PSCs can have different designs, like the standard n–i–p, inverted p–i–n, or mesoscopic structures with scaffold layers (Figure 4).^142,143^

**Figure 4:**
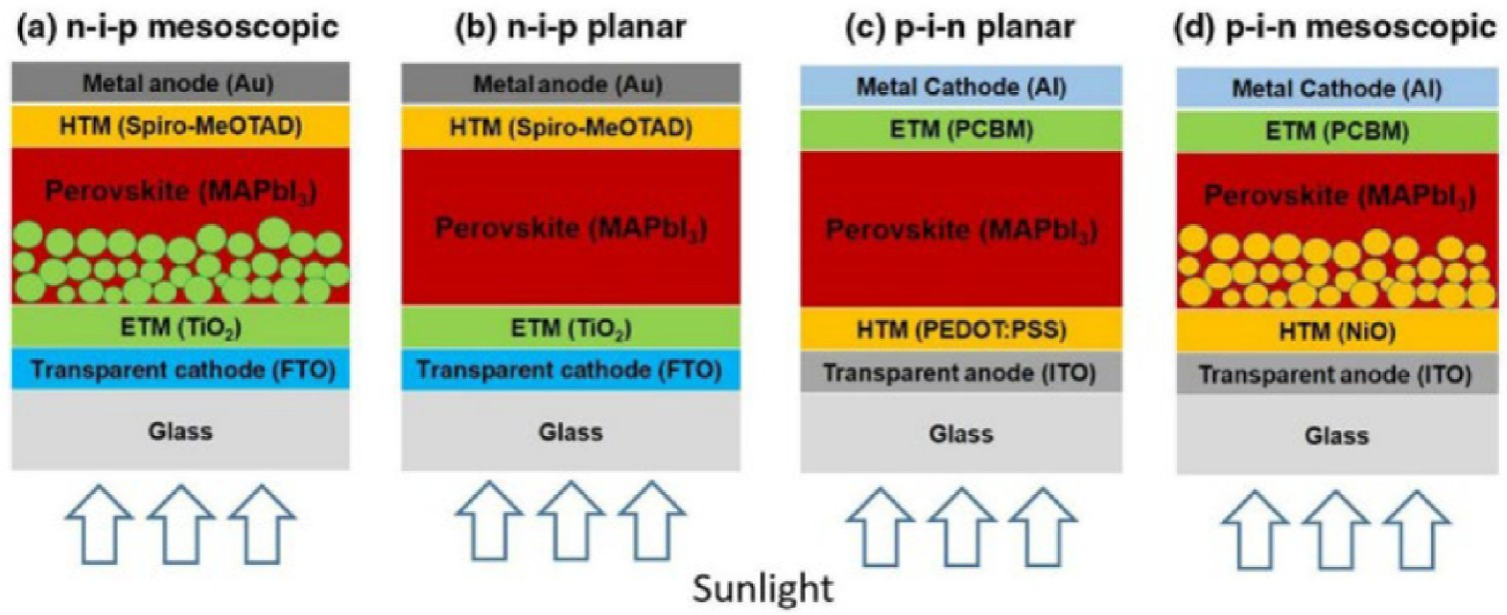
Four-layered configurations of PSCs: (a) n-i-p mesoscopic, (b) n-i-p planar, (c) p-i-n planar, and (d) p-i-n mesoscopic, Reproduced with Permission, Copyright,[Bibr ref143] 2022.

Optimizing each layer in PSCs and their interfaces is necessary for reducing recombination losses, optimizing charge extraction, and increasing device lifespan. The comprehensive analysis of their interface architecture is presented in the following section.

##### 3.1.1.5 Interface Engineering

**Hole Transport Layer (HTL) in PSCs:** When electrons are blocked to prevent recombination, the HTL in PSCs aids in the transmission of holes from the perovskite layer to the anode. It culminates in perovskite deterioration and ion migration, diminishing efficiency over time. In conventional n-i-p perovskite solar cells, Spiro-OMeTAD is a commonly utilized hole transport layer due to its favorable energy level alignment with lead halide perovskites. Materials or PSCs infused with Lithium bis­(trifluoromethanesulfonyl)­imide (Li-TFSI) and 4-tert-Butylpyridine (tBP) to augment conductivity; nevertheless, these dopants are hygroscopic and thermally unstable. A dopant-free polymer PTAA is utilized in inverted p-i-n perovskite solar cells (PSCs). Poly­(triaryl amine) (PTAA) reveals a commendable energy level, possesses water resistance, and produces consistent films. This additionally aids in diminishing recombination and enhancing device stability. PTAA does not need harmful dopants, so it is more stable under light and heat. However, it has lower hole mobility and sticks poorly to perovskite, so surface treatments like Self-assembled monolayers (SAMs), oxygen plasma, or added interlayers (NiOx, graphene oxide) are used to fix this. PSCs with PTAA show efficiencies over 24% with better moisture and heat resistance, especially in flexible and tandem cells (Figure 5).^144^

**Figure 5:**
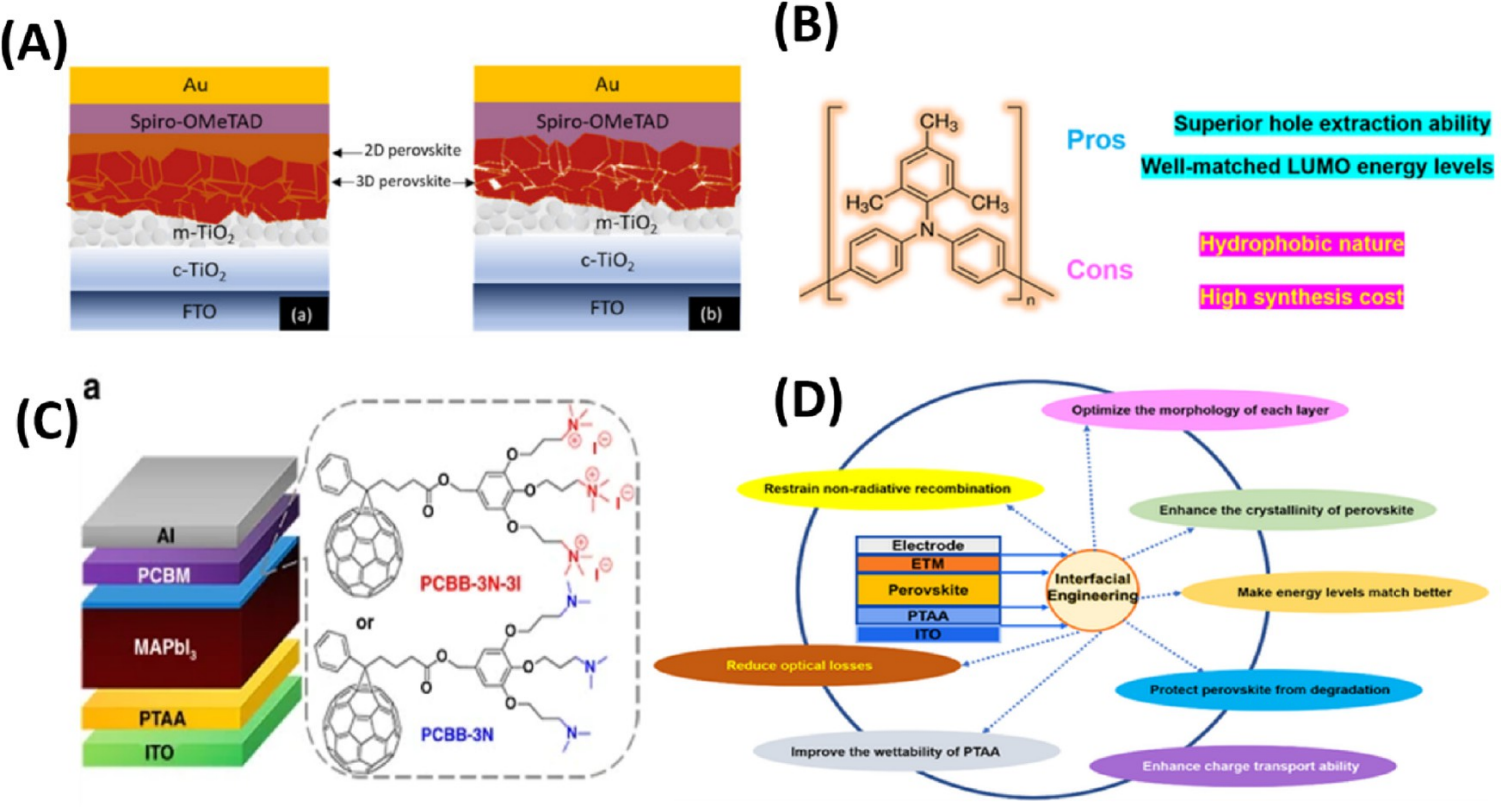
(A) HTLs in PSCs with n-i-p architecture. (B) The pros and cons chemical structure of PTAA. (C) Schematic structure of ITO/PTAA/MAPbI_3_/PCBM/Al, both without and with PCBB-3N-3I/PCBB-3N as SPL, (D) PTAA-IPSC interfaces interfacial engineering, Reproduced with permission, Copyright, [Bibr ref143],[Bibr ref144] 2022, 2024.

Self-assembled monolayers (SAMs) have emerged as one of the most promising and widely adopted classes of hole transport layers (HTLs) for high-efficiency and stable PSCs.^145–149^ Unlike conventional organic HTLs such as PEDOT: PSS, PTAA, or Spiro-OMeTAD, which often suffer from interfacial energy mismatch, hygroscopicity, and poor long-term stability, SAMs form a highly ordered, covalently bonded monolayer at the perovskite/electrode interface, enabling superior charge extraction and interfacial passivation. The use of carbazole-, triphenylamine-, or phosphonic acid-based SAMs has been demonstrated to significantly reduce interfacial recombination, minimize energy-level offsets, and improve device operational stability under continuous illumination.^150–152^ Furthermore, SAM-based HTLs enable scalable, low-temperature, and solution-processable fabrication, which aligns well with industrially compatible roll-to-roll or slot-die printing approaches. This standard shift toward SAMs reflects a range to moves toward interface-engineered and dopant-free architectures that combine simplicity with long-term durability, thus representing a critical direction in next-generation perovskite device engineering.^153–166^ Although HTL optimization improves hole extraction and interfacial stability, reported studies also show that electron collection and suppression of interfacial recombination are equally dependent on the properties of the ETL. Table 2 provides recent research and the comparative changes using PTAA, or Spiro-OMeTAD organic layer.

**Table 2:** Comparative Performance of Perovskite Solar Cells using PTAA and Spiro-OMeTad as the Hole Transport Layer (HTL) in terms of Device Configuration, Open-Circuit Voltage (*V*_OC_), Fill Factor (FF), and Power Conversion Efficiency (PCE)

S.No	Device Configuration	HTL	*V*_OC_ (V)	FF	PCE%	Refs
1	ITO/PEDOT:PSS/Perovskite/Spiro-OMeTAD:P4VP/Au	P4VP	1.129	0.787	20.84	^153^
2	FTO/c-TiO_2_/Cs_0.05_FA_0.81_MA_0.14_PbI_2.55_Br_0.45_/Spiro-OMeTAD:NPC/Au	NPC	1.06	0.76	18.51	^154^
3	ITO/SnO_2_–KCl/CsFAMA/Spiro-OMeTAD:Sb_2_S_3_/Au	Sb_2_S_3_	1.132	0.79	22.13	^155^
4	FTO/TiO_2_/Cs_0.05_MA_0.1_FA_0.85_Pb(I_0.97_Br_0.03_)_3_/Spiro-OMeTAD:CoCN_2_/Au	Co(III)-CN	1.138	0.795	23.01	^156^
5	FTO/SnO_2_/(FAPbI_3_)_0.95_(MAPbBr_3_)_0.05_/CQDs/Spiro-OMeTAD/Ag	CQDs	1.064	0.799	17.92	
6	ITO/SnO_2_/CsFAMA/PbSO_4_(PbO)_4_-Spiro-OMeTAD/Au	PbSO_4_(PbO)_4_ QDs	1.142	0.80	22.66	^160^
7	ITO/SnO_2_/Perovskite/SnS-Spiro-OMeTAD/Au	SnS	1.17	0.807	22.59	^161^
8	ITO/PEDOT:PSS/MAPbI_3_/PCBM/Rhodamine/Ag	Urea	1.03	0.809	18.80	^162^
9	ITO/SnO_2_/FA_0.4_MA_0.6_PbI_2.8_Br_0.2_/WO_3_/PEDOT:PSS/MoO_3_/Ag	WO_3_	1.03	0.648	15.10	^163^
10	ITO/D-PEDOT:PSS/MAPbI_3–x_Cl_*x*_/C_60_/BCP/Ag	Diluted	1.08	0.804	17.85	^164^
11	ITO/SC-PEDOT:PSS/MAPbI_3–x_Cl_*x*_/PCBM/BCP/Ag	Citrate	1.134	0.750	18.39	^165^

**Electron Transport Layer (ETL) in PSCs: TiO_2_ vs SnO_2_:** The ETL in PSCs helps in two essential functions. First, this involves extracting photoexcited electrons from the perovskite absorber and transporting them efficiently to the cathode, while acting as a barrier against holes to minimize interfacial charge recombination. Traditionally, TiO_2_ has been the most widely used ETL due to its favorable conduction band position (4.0 eV), wide bandgap (∼3.2 eV), chemical stability, and compatibility with mesoscopic structures. It forms a blocking layer or mesoporous scaffold that enhances charge storage or gathering. However, TiO_2_ suffers from significant drawbacks, including UV-light-induced photocatalytic activity, which leads to the decomposition of the perovskite layer, especially under ambient conditions, and high-temperature processing (≈450–500 °C) that is incompatible with flexible or low-cost substrates. Furthermore, these kinds of PSCs provide low electron mobility and high trap-state density, which often resulting in increased hysteresis and reduced PCE in devices.

Despite these limitations, SnO_2_ (tin oxide) has appeared as a superior alternative ETL due to its high electron mobility, wide bandgap around 4.2 eV, and ability to be processed at low temperatures less than 150 °C, making it highly suitable for flexible, tandem, and roll-to-roll processed PSCs. But, SnO_2_ exhibits better transparency in the visible region, reduced photocatalytic behavior, and lower defect density at the interface. These properties result in significantly reduced hysteresis and enhanced open-circuit voltage (Voc) and fill factor (FF). In contrast to TiO_2_, SnO_2_ exhibits exceptional stability under UV light and prolongs the operating lifetimes of PSCs. Electron extraction efficiency and interface contact are improved by interface passivation techniques such as Mg^2+^, Cl^–^, or K^+^ ion doping and interface manipulation with fullerene-like compounds or self-assembled molecules. Because of its high electron mobility, low processing temperature (at or below 150 °C), and economic feasibility, SnO_2_ has attracted attention as an electron transport layer (ETL). However, it often encounters lattice mismatch and oxygen vacancies upon interaction with CsPbI_3_-_*x*_Br_*x*_, leading to nonradiative recombination losses. Conversely, the widely utilized TiO_2_ electron transport layer necessitates high-temperature processing, rendering it inappropriate for flexible or tandem perovskite solar cells due to issues such as surface flaws and inadequate homogeneity.^167–189^ While advances in ETL design significantly enhance charge extraction and reduce energy losses, recent literature indicates that further efficiency gains require architectural innovations beyond single-junction devices. This motivates the transition toward tandem perovskite architectures. Table 3 provides recent reported advances in the field of PSCs and their comparative power conversion efficiency.

**Table 3:** Recent Advances in Perovskite Solar Cells (PSCs) using Various Electron Transport Layers (ETLs) and Their Comparative Device Performance in terms of Open-Circuit Voltage (*V*_OC_), Fill Factor (FF), and Power Conversion Efficiency (PCE)

S.No.	Device Configuration	ETL	*V*_OC_ (V)	FF	PCE (%)	Refs
1	FTO/c-TiO_2_/Li-m-TiO_2_/MAPbI_3_/Spiro-OMeTAD/Au	Li	1.101	0.699	17.59	^167^
2	FTO/Mg-TiO_2_/Perovskite/Spiro-OMeTAD/Au	Mg	1.08	0.609	14.65	^168^
3	FTO/Zn-TiO_2_/MAPbI_3_/Spiro-OMeTAD/Au	Zn	1.10	0.734	17.60	^169^
4	FTO/Zn-TiO_2_ NAs/(FAPbI_3_)_0.87_(MAPbBr_3_)_0.13_/CuSCN/Carbon	Zn	0.956	0.679	14.45	^170^
5	FTO/EuAc_3_-c-TiO_2_/CsPbI_3_/P3HT/Au	EuAc_3_	1.1	0.77	17.92	^171^
6	FTO/Nb-TiO_2_/FA_0.79_MA_0.16_Cs_0.05_Pb(Br_X_I_1–X_)_3_/Spiro-OMeTAD/Au	Nb	1.12	0.78	21.30	^172^
7	FTO/Ce-TiO_2_/MAPbI_3_/Spiro-OMeTAD/Ag	Ce	1.07	0.69	16.18	^173^
8	FTO/Zr-TiO_2_/MAPbI_3_/Spiro-OMeTAD/Ag	Zr	0.92	0.567	12.35	^174^
9	FTO/Zr-TiO_2_/MAPbI_3_/Spiro-OMeTAD/Au	Zr	1.076	0.716	18.16	^175^
10	FTO/Ta-TiO_2_/Cs_0.1_(FA_0.83_MA_0.17_)_0.9_Pb(I_0.83_Br_0.17_)_3_/Spiro-OMeTAD/Ag	Ta	1.13	0.77	19.62	^176^
11	FTO/c-TiO_2_/GQDs-mTiO_2_/Cs_0.05_(FA_0.83_MA_0.17_)_0.95_ Pb(I_0.83_Br_0.17_)_3_/Spiro-OMeTAD/Au	GQDs	0.97	0.63	14.36	^177^
12	ITO/Ga-SnO_2_/(FAPbI_3_)_*x*_(MAPbBr_3_)_1‑x_/Spiro-OMeTAD/Ag	Ga	1.068	0.714	18.18	^178^
13	FTO/Ga-SnO_*x*_/CsPbBr_3_/Carbon	Ga	1.311	0.602	5.98	^179^
14	ITO/Ta-SnO_2_/Perovskite/Spiro-OMeTAD/Au	Ta	1.161	0.786	20.8	^180^
15	FTO/Nb-SnO_2_/CsPbBr_3_/Carbon	Nb	1.31	0.731	8.54	^182^
16	FTO/Zn-SnO_2_/CsPbBr_3_/CuPc/Carbon	Zn	1.098	0.692	17.78	^183^
17	Li-FTO/SnO_2_/Al_2_O_3_/MAPbI_3_/Carbon	Li	0.76	0.59	10.01	^184^
18	FTO/Zr/F-SnO_2_/Perovskite/Spiro-OMeTAD/Au	Zr/F	1.105	0.712	19.19	^185^
19	FTO/c-TiO_2_/Li-m-TiO_2_/MAPbI_3_/Spiro-OMeTAD/Au	Li	1.101	0.699	17.59	^167^
20	FTO/Mg-TiO_2_/Perovskite/Spiro-OMeTAD/Au	Mg	1.08	0.609	14.65	^168^
21	FTO/Zn-TiO_2_/MAPbI_3_/Spiro-OMeTAD/Au	Zn	1.10	0.734	17.60	^169^
22	FTO/Zn-TiO_2_ NAs/(FAPbI_3_)_0.87_(MAPbBr_3_)_0.13_/CuSCN/Carbon	Zn	0.956	0.679	14.45	^170^
23	FTO/EuAc_3_-c-TiO_2_/CsPbI_3_/P3HT/Au	EuAc_3_	1.1	0.77	17.92	^171^
24	FTO/Nb-TiO_2_/FA_0.79_MA_0.16_Cs_0.05_Pb(Br_X_I_1–X_)_3_/Spiro-OMeTAD/Au	Nb	1.12	0.78	21.30	^172^
25	FTO/Ce-TiO_2_/MAPbI_3_/Spiro-OMeTAD/Ag	Ce	1.07	0.69	16.18	^173^
26	FTO/Zr-TiO_2_/MAPbI_3_/Spiro-OMeTAD/Ag	Zr	0.92	0.567	12.35	^174^
27	FTO/Zr-TiO_2_/MAPbI_3_/Spiro-OMeTAD/Au	Zr	1.076	0.716	18.16	^175^
28	FTO/Ta-TiO_2_/Cs_0.1_(FA_0.83_MA_0.17_)_0.9_Pb(I_0.83_Br_0.17_)_3_/Spiro-OMeTAD/Ag	Ta	1.13	0.77	19.62	^176^
29	FTO/c-TiO_2_/GQDs-mTiO_2_/Cs_0.05_(FA_0.83_MA_0.17_)_0.95_ Pb(I_0.83_Br_0.17_)_3_/Spiro-OMeTAD/Au	GQDs	0.97	0.63	14.36	^177^
30	ITO/Ga-SnO_2_/(FAPbI_3_)_*x*_(MAPbBr_3_)_1‑x_/Spiro-OMeTAD/Ag	Ga	1.068	0.714	18.18	^178^
31	FTO/Ga-SnO_*x*_/CsPbBr_3_/Carbon	Ga	1.311	0.602	5.98	^179^
32	ITO/Ta-SnO_2_/Perovskite/Spiro-OMeTAD/Au	Ta	1.161	0.786	20.8	^180^
33	FTO/Nb-SnO_2_/CsPbBr_3_/Carbon	Nb	1.31	0.731	8.54	^182^
34	FTO/Zn-SnO_2_/CsPbBr_3_/CuPc/Carbon	Zn	1.098	0.692	17.78	^183^
35	Li-FTO/SnO_2_/Al_2_O_3_/MAPbI_3_/Carbon	Li	0.76	0.59	10.01	^184^
36	FTO/Zr/F-SnO_2_/Perovskite/Spiro-OMeTAD/Au	Zr/F	1.105	0.712	19.19	^185^
37	ITO/KF-SnO_2_/CsPbI_2_Br/Spiro-OMeTAD/MoO_3_/Au	KF	1.31	0.792	15.39	^186^
38	FTO/Cl-SnO_2_/Perovskite/Spiro-OMeTAD/Au	Cl	1.07	0.73	18.94	^187^
39	ITO/NH_4_Cl-L-SnO_2_/NH_4_Cl–H–SnO_2_/Perovskite/PEAI/Spiro-OMeTAD/Au	NH_4_Cl	1.208	0.762	21.75	^188^
40	ITO/GQDs-SnO_2_/MAFAPbI_3_Cl_3‑x_/Spiro-OMeTAD/Ag	GQDs	1.11	0.78	21.10	^189^

As research focused to resolve these challenges one of researcher Lin et al.^190^ developed a bilayer ETL combining SnO_2_ via electron beam evaporation and TiO_2_ via hydrothermal growth. This configuration improved charge extraction, reduced recombination, and boosted overall device performance. The bilayer PSC showed a PCE of 11.48%, notably higher than the 8.09% achieved with single-layer ETLs, and delivered a ∼15% higher fill factor. This work highlights the SnO_2_/TiO_2_ bilayer as a promising ETL design for efficient and stable all-inorganic PSCs. Three different kinds of SnO_2_/TiO_2_ bilayer ETLs were made to examine how the ETLs affected the performance of PSCs: SnO_2_, TiO_2_, and SnO_2_/TiO_2_. Figure 6A­(a–c) shows the structural diagram of the PSC using these ETLs. Twenty perovskite solar cells were made under each condition, and the results were examined under light to examine the variations in photovoltaic performance of the various ETLs. The champion device’s forward and backward scanned J-V curves under the AM1.5 solar simulator are shown in Figure 6B­(a). In comparison to cells based on single-layer TiO_2_ ETL and single-layer SnO_2_ ETL, the stability test findings show that the perovskite solar cells based on the SnO_2_/TiO_2_ bilayer ETL have greater electron extraction capabilities and superior stability overall. The results of the humid air storage stability tests for PSCs based on the monolayer TiO_2_ ETL, monolayer SnO_2_ ETL, and SnO_2_/TiO_2_ bilayer ETL are shown in Figure 6B­(b). For 60 h, the cells were exposed to a relative humidity of 20–30%. When compared to cells based on monolayer TiO_2_ ETL and monolayer SnO_2_ ETL, it is evident that PSCs based on SnO_2_/TiO_2_ bilayer ETLs show superior stability in storage.^190^

**Figure 6:**
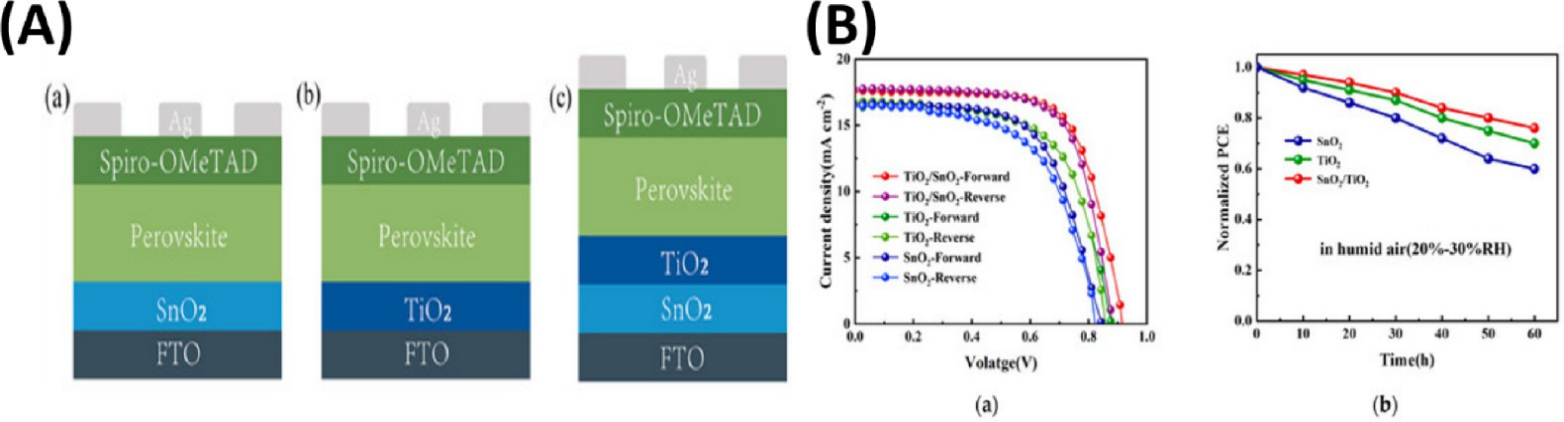
(A) schematic design of PSC device constructions based on a- SnO_2_, b- TiO_2_, and c- SnO_2_/TiO_2_. (B) a- The PSCs’ champion *J*–*V* curves with various ETL kinds. b- The stability test for the device’s lifespan (20–30% relative humidity for 60 h). Reproduced with permission, Copyright,[Bibr ref190] 2023.

**Tandem Solar Cells:** TSCs have brought a big step forward in the journey to commercialize PSCs. These tandem structures combine two different types of solar cells—one on top of the other—to use sunlight more efficiently. While the process of making tandem cells involves extra steps, the goal is to achieve higher PCE and better long-term stability. In PSCs, layers separate the top and bottom cells, must be stable and help reduce energy loss, which ensure this in both solar cells in tandem structures and also with their intermediate layers. Research showed that adding a thin layer of silver (Ag) nanostructures made by solution-processing helps lower recombination losses in perovskite solar cells (PSCs). Tandem solar cells (TSCs) combining wide and low bandgap materials absorb more sunlight and reach higher efficiencies.^191^ Tunable band gaps of PSCs suit this well and can pair with materials like organic compounds, other perovskites, silicon (Si), or CIGS. Perovskite/Si tandem cells have achieved certified efficiencies above 33.7%, exceeding the single-junction Shockley–Queisser limit of 33%. Still, challenges remain, especially the poor perovskite layer quality formed when deposited directly on the silicon bottom cell’s rough surface before adding the electrode. It is challenging to produce consistent, high-quality perovskite films with this configuration.^192^ Researchers have suggested several tactics, including passivating the perovskite surface to minimize defects, altering the precursor solution to promote crystal development, and adding a buffer layer to protect the perovskite. tandem cells is that the photocurrent depends on how well the top and bottom cells are matched. If one cell produces less current than the other, the whole device loses efficiency. Other variables that may impact performance include the resistance of the transparency electrodes, the HTL, and the ETL. Thermalization losses, which happen when high-energy photons release excess energy as heat, are successfully reduced using tandem solar cells. Through the integration of two absorber layers with different band gaps, tandem cells can improve the transformation of sunlight into energy. Three main configurations are typically used in the manufacturing of tandem devices: In the two-terminal (2-terminal) design, the top and bottom cells share a single circuit and are connected in series. Performance is limited by this arrangement since both cells must supply the same current, especially in situations with fluctuating light levels. Second, each cell may control its own electrical contacts thanks to the four-terminal (4-Terminal) arrangement. This calls for more transparent layers, which could increase radiation losses and reduce total power generation. Third, as illustrated in Figure 7, the three-terminal (3-Terminal) architecture, it is a more recent innovation that combines the advantages of 2T and 4T. It allows optical connection but electrical separation, helping to avoid both current mismatch and excessive optical losses.^193^ Recent results show that the 3T design can outperform the others by improving external quantum efficiency (EQE) and power output across different lighting conditions. Despite the remarkable efficiency gains achieved with tandem architectures, recent literature underscores that their practical impact remains constrained by persistent thermal, environmental, and interfacial degradation processes. Accordingly, the following section critically examines the stability challenges that must be addressed to enable reliable and scalable perovskite photovoltaic technologies.

**Figure 7:**
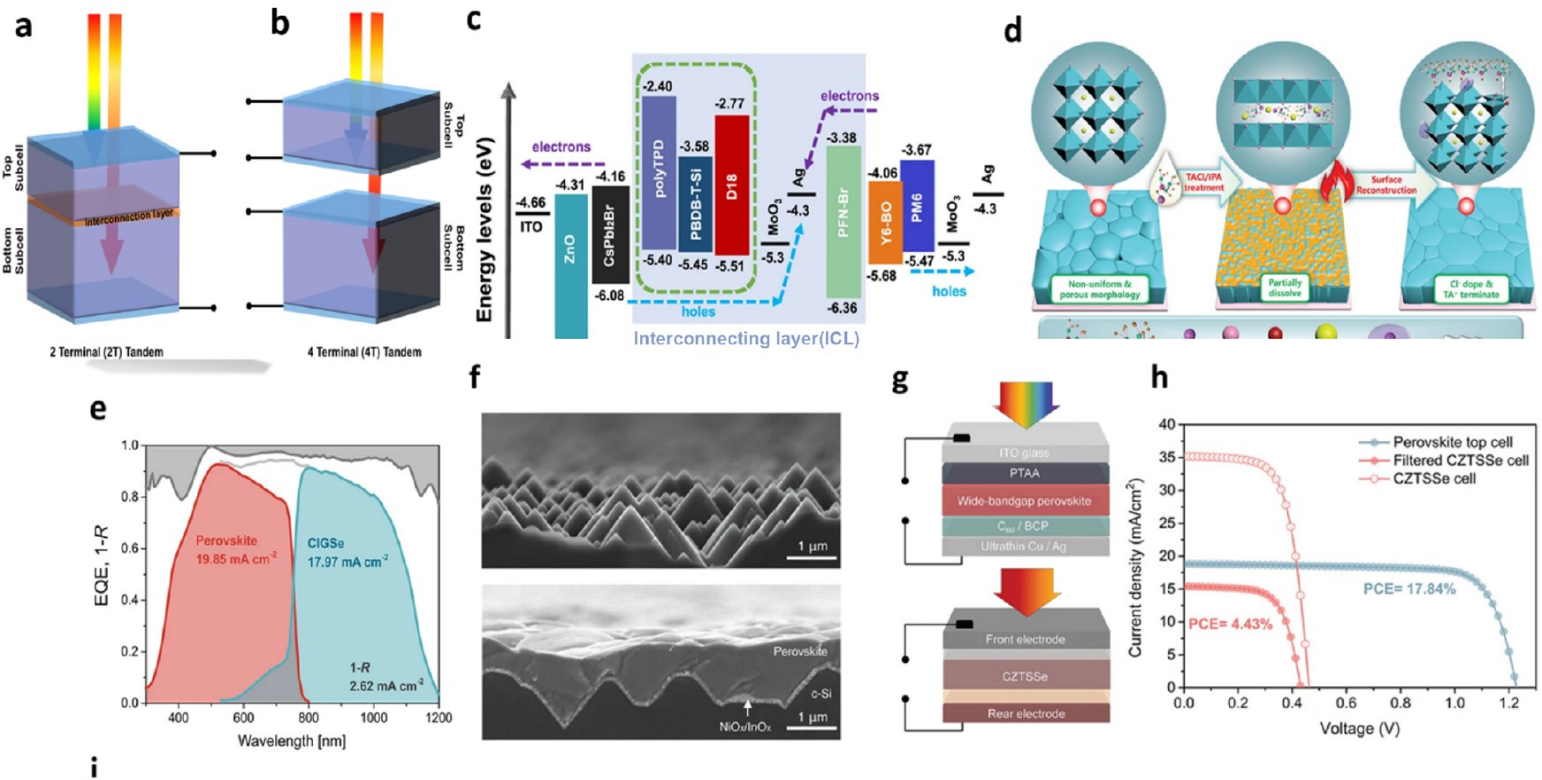
(a, b) The two most popular tandem designs (2T and 4T) and their structures, Reproduced with permission, Copyright,[Bibr ref193] 2021. (c) An inorganic perovskite/organic tandem solar cell (TSC) energy-level diagram. Reproduced with permission, Copyright,[Bibr ref194] 2022. (d) Diagrammatic representation of the surface reconstruction (SR) processes that are synergistically triggered by IPA and trimethylammonium chloride (TACl). Reproduced with permission, Copyright,[Bibr ref195] 2019. (e) The total reflection spectra, represented as 1-R, and the individual subcell EQE spectra of a NiOx/PTAA TSCs are measured. Red, blue, and gray lines and regions indicate perovskite, CIGSe, and 1-R spectra. Additionally displayed are integrated photocurrents and reflection losses derived from the 1-R and EQE spectrum values. Reproduced with permission, Copyright,[Bibr ref196] 2020. (f) Cross-sectional SEM pictures of a textured c-Si with a typical pyramid size of 2 μm (top) and matching substrates coated with perovskite crystals that have been solution-processed (bottom). Reproduced with permission, Copyright,[Bibr ref197] 2022. (g) Perovskite/CZTSSe TSCs device architecture. (h) *J*–*V* curves for several kinds of tandem and subcells based on CZTSSe and perovskites. Reproduced with permission, Copyright,[Bibr ref198] 2019.

#### 3.1.2 Stability

Perovskite solar cells (PSCs) face several important stability challenges that limit their lifespan and performance. These problems mostly arise due to exposure to oxygen, moisture, ultraviolet (UV) and visible light, and high temperatures.^199^ Since PSCs are intended for outdoor use, they must be strong enough to handle tough environmental conditions. Unlike traditional crystalline silicon solar cells, which can last for more than 30 years, the stability of PSCs reported in the literature is much lower—typically up to 10,000 h (about 1 year),^201^ 5,200 h,^202^ or 4,000 h.^200^ The stability of PSCs can be enhanced by comprehending their degradation over time. PSCs must endure accelerated aging tests that simulate real-world conditions, including thermal and humidity cycles, ultraviolet light exposure, and continuous light exposure at temperatures of 60 or 85 °C.^207,210–213,218,335–337,339,352,353,366,385^ Through the simulation of their outdoor performance over time, these studies allow the evaluation of competitiveness of PSCs in comparison to existing solar technologies. Usually, tests take 1,000 h to complete. According to international standards, crystalline silicon cells can only lose 5% of their efficiency over the same time span, whereas thin-film solar cells can lose up to 10%.^203^ After 500 h, many PSCs only maintain around 80% of their initial efficiency, even with the inclusion of additional protective coatings. Numerous stability tests in the literature have been carried out in less demanding settings, like dark storage or light exposure at 25 or 60 °C, which could not accurately reflect exterior performance.^204^ The toxicity and safety risks associated with lead (Pb) inclusion in PSCs are a significant worry.^205^

For the large-scale manufacturing of PSCs, researchers continue to investigate the best and most dependable fabrication techniques. Researchers are investigating a variety of approaches to address these issues and increase material stability, including compositional engineering to change the chemical composition for increased stability, interfacial engineering to maximize the relationship between the various layers in the solar cell, and cross-linking additives to reinforce the crystal structure.^206^ The goal of these research initiatives is to increase perovskite solar cells’ (PSCs) dependability, effectiveness, and affordability. Recent research has concentrated on creating thorough stabilization techniques to fortify PSCs’ structure and interfaces in light of the numerous variables that can contribute to degradation. Inorganic charge transport layers like TiO_2_ and SnO_2_ are used to improve chemical stability in addition to additive engineering, which employs molecular or ionic passivators to stop defect development and ion migration. Each of these techniques contributes significantly to enhancing the inherent robustness and operational stability of perovskite devices, as covered in the sections that follow.

#### 3.1.3 Carrier Transport Materials and Device Stability

One of the biggest obstacles to increasing the stability of perovskite solar cells (PSCs) is still the search for appropriate charge transport materials. Finding efficient charge transfer techniques has been the subject of numerous investigations. Early devices that used Poly­(3-hexylthiophene) (P3HT) in conjunction with CH_3_NH_3_PbI_2_Br had stable operation for up to 250 h with moderate efficiencies of about 10%.^208,209^ The potential for improving durability has been demonstrated by water-resistant hole transport materials (HTMs). It has also been discovered that some dopants, including iridium complexes, enhance stability over the long run. LiTFSI and other additions, on the other hand, are helpful for boosting HTM conductivity but tend to be corrosive or readily absorb moisture, which causes instability. Device stability has increased by substituting more stable dopants, like those based on spiro, for these sensitive ones.

HTM-free perovskite solar cells were initially created to solve the instability problems with traditional hole-transport materials, however reported devices continue to have low efficiency and short operational lifespans. Recent research on carbon-based PSCs shows that the choice of HTM has a significant impact on long-term stability and performance. For instance, after 500 h of ambient aging, PTAA-based devices only maintain about 35% of their initial efficiency, but Spiro-OMeTAD-based Carbon based-PSCs can attain PCEs of up to 19.3%. Even CuSCN, which provides better stability, only maintains about 60% of its initial performance within the same time frame. These results show that stability issues cannot be completely resolved by removing HTMs alone, and electrode selection and interfacial engineering continue to be crucial factors in device longevity.^214^ The lifespan of the gadget is also impacted by the metal electrode selection. Although gold electrodes are very stable and perform well, their exorbitant cost prevents large-scale manufacture. Despite the great stability and outstanding electrical performance of gold electrodes, it is crucial to remember that under operating conditions, Au can experience ion migration and interdiffusion, which could result in degradation pathways and long-term instability in perovskite solar cells. Although less expensive materials, such as silver pastes, have undergone testing, their lack of environmental resilience makes them less suitable for industrial usage. According to studies, employing protective HTM layers and hydrophobic electrodes can improve overall stability and moisture resistance.^214,215^

Remaining stability problems have been addressed in a number of ways, such as changing the composition of the perovskite by adding mixed halides such as CH_3_NH_3_PbI_2_Br to increase structural stability. This strategy might, however, marginally increase the bandgap, which would lower efficiency.^157,216,217^ Additional documented techniques include the use of UV filters, the addition of buffer layers, the doping of TiO_2_ layers, the use of different HTMs and electrodes, and structural modifications to the perovskite absorber. Thermal deterioration continues to be a significant obstacle for long-term operation despite recent advancements in encapsulating technology.

#### 3.1.4 Structural and Thermal Instability

Hybrid perovskite solar cells possess characteristics that affect their stability and efficiency, linked to their structural properties. The transition of the perovskite structure from cubic to tetragonal negatively affects light absorption and power generation, resulting in reduced solar cell efficiency. The Goldschmidt tolerance factor, based on the ionic radii of cations and anions, plays a crucial role in determining the stability of the multidimensional perovskite structure. The optimal tolerance factor (t) for stable three-dimensional cubic perovskite structures ranges from 0.9 to 1. Mixed cation perovskites, such as Formamidinium cesium (FA-Cs) PbI_3_, demonstrate a tolerance factor between 0.94 and 0.98, which contributes to their stability.^219^ MAPbI_3_ (methylammonium lead iodide) undergoes a phase transition from tetragonal to cubic at 55 °C, whereas similar halide perovskites like MAPbBr_3_ and MAPbCl_3_ exhibit stability between 40 and 85 °C.^219^ Thermal examination techniques, including thermogravimetric analysis (TGA), indicate that the organic components of MAPbX_3_ decompose into HI and CH_3_NH_2_, leading to further degradation.^220^ The minimum development energy of MAPbI_3_-_*x*_Cl_*x*_, which is comparable to the thermal energy at 85 °C, suggests the material’s vulnerability to degradation.^221^ Another finding suggests that annealing, used to improve crystallinity, may accelerate degradation. FAPbI_3_ materials demonstrate greater thermal stability compared to MAPbI_3_.^221^ The use of carbon-based materials, such as graphene, carbon nanotubes (CNTs), and carbon fibers, significantly improves thermal endurance.^199,222^ Goldschmidt et al. noted that single-component perovskites show significant performance; however, MAPbI_3_ displays insufficient thermal stability.^223^ FAPbI_3_ demonstrates a favorable bandgap; however, it faces challenges associated with phase transitions that undermine its stability in the desired α-phase. Elevated temperatures generally result in the degradation of hybrid perovskites, which consist of organic and inorganic elements, yielding unwanted byproducts such as organic vapors and lead halides. This leads to the degradation of the three-dimensional structure and a reduction in device performance.

CsPbI_3_, a fully inorganic perovskite, exhibits enhanced thermal stability owing to the lack of volatile organic components like methylammonium (MA) or formamidinium (FA). Thus, CsPbI_3_ exhibits enhanced thermal stability compared to hybrid perovskites.^223^ To enhance stability and optimize properties, researchers frequently modify the ions located at the A, B, and X sites within the ABX_3_ perovskite structure. The structural stability of perovskites depends on the octahedral factor (μ) and Goldschmidt’s tolerance factor (t).^224^

$$\mu =\frac{r_{B}}{r_{X}}$$

$$t=\frac{r_{A+}r_{X}}{\sqrt{2}\left(r_{B+}r_{X}\right)}$$

Here, $r_{A}$ , $r_{B}$ , and $r_{X}$ represent the ionic radii of the A-site cation, B-site cation, and X-site anion, respectively. Research shows that perovskites are stable when 0.813 < *t* < 1.1070, as well as 0.414–0.732 for a stable BX_6_ octahedral coordination. A good cubic structure is usually seen when *t* is between 0.8 and 1.^225,226^ The perfect stability occurs at *t* = 1. Deviating from this value causes the structure to bend or distort.

Figure 2A­(a) shows how different *t* values affect the structure. For hybrid perovskites, *t* < 0.8 often leads to an orthorhombic shape, while 0.8< *t* < 1 gives a stable cubic form. If *t* > 1, the structure may turn hexagonal, often forming layered versions like the Ruddlesden–Popper (RP) phase. If *t* < 0.7, a nonperovskite structure usually forms. Once the intrinsic structural and thermal limits are recognized, another dimension of degradation becomes apparent: the perovskite framework interacts readily with ambient species. Evidence from environmental-exposure studies shows that oxygen and moisture penetrate and destabilize the lattice even under mild conditions. Hence, environmental instability forms the next critical area of focus.

##### 3.1.4.1 Oxygen and Moisture

Oxygen and moisture are one of the most serious challenges for the stability of PSCs. Oxygen reacts with and oxidizes the organic components in perovskite, while moisture causes structural damage by breaking down the material. Some or researchers reported that PSCs are often stored in dark and dry environments, since light combined with moisture can trigger photo-oxidation.^227^ The hygroscopic properties of amine salts utilized in PSCs result in moisture absorption from the atmosphere, causing the development of hydrate phases that compromise the structural integrity of the device.^228^ The presence of heat and humidity accelerates degradation.^229^ Proposed decomposition pathways in the presence of moisture and oxygen include:^230^ for instance,

$${\mathrm{CH}}_{3}{\mathrm{NH}}_{3}{\mathrm{PbI}}_{3}\to {\mathrm{PbI}}_{2}+{\mathrm{CH}}_{3}{\mathrm{NH}}_{2}+\mathrm{HI}$$

$$3{\mathrm{CH}}_{3}{\mathrm{NH}}_{3^{+}}\to {3\mathrm{CH}}_{3}{\mathrm{NH}}_{2}+3H^{+}$$

$${\mathrm{4HI + O}}_{2}\to {\mathrm{2I}}_{2}{\mathrm{+ 2H}}_{2}O$$

$$\mathrm{2HI}\to I_{2}+H_{2}$$

Each of these steps lead to the degradation of the perovskite active layer, making moisture and oxygen considerable threats to the device’s enduring stability. Investigations indicate that metal halide perovskites demonstrate significant stability when kept in dark conditions, implying low deterioration without light exposure. However, in the presence of light, MAPbI_3_ perovskite swiftly commences decomposition. Oxygen has a crucial role in its degradation, especially when exposed to light. Figure 2B­(a) illustrates that the degradation of the MAPbI_3_ surface transpires progressively. Initially, light excites electrons in MAPbI3, which then interact with neighboring oxygen molecules (Step I), leading to the generation of superoxide (O_2_^–^). The O_2_^–^ engages with the [CH_3_NH_3_] ^+^ ions and lead (Pb) atoms on the MAI-terminated surface, leading to the production of water and Pb­(OH)_2_, which further exposes the surface (Step II). The degradation byproducts obstruct further oxygen infiltration; however, water continues to permeate the film, resulting in the hydration and eventual breakdown of the inner perovskite over time (Step III).^231–233^

Oxygen not only directly affects perovskite but also interacts with TiO_2_, a substance commonly utilized as a charge transfer layer. TiO_2_ interacts with oxygen, leading to the generation of supplementary O_2_^–^ and expediting perovskite degradation.^234,235^ To protect electronics from oxygen degradation, researchers have assessed a two-layer (2D/3D) perovskite structure. Shao et al. revealed that the inclusion of a small amount of 2D tin-based perovskite into the standard 3D FASnI_3_ improved crystal quality, structural stability, and defect resilience. Devices made from this hybrid material demonstrated enhanced performance and durability under ordinary room conditions. While the 2*D*/3D version maintained 59% of its original power, the 3D-only device malfunctioned after 76 h. Their improved stability stemmed from more resilient crystals and greater moisture resistance.^236^ Although oxygen can adversely affect perovskite materials by interacting with organic cations such as MA^+^ and FA^+^, it can also serve to passivate faults and diminish energy losses. Oxygen can occupy halide vacancies, so diminishing nonradiative recombination and enhancing performance.^63^ Figure 2B­(b) explains this “photo-brightening” effect—after exposure to light, O^2^, and humidity, defect states decrease, making light emission stronger by increasing radiative recombination and reducing nonradiative losses. In detail, Figure 2B­(ci-ii) shows untreated MAPbI_3_ with surface defects. After being exposed to light and O_2_, superoxide species form and reduce surface defects, causing photobrightening. This change is temporary and can be reversed if the sample is kept in the dark. Figure 2B­(ciii) shows what happens when water and O_2_ are both present-surface defects disappear almost completely, and a thin degraded layer forms. This degraded layer acts as a barrier, holding back oxygen and reducing ion movement inside the film.^237^ Beyond the environmental effect, PSCs’ absorbers undergo additional deterioration under operational illumination. Photoexcitation initiates bond cleavage, halide redistribution, and surface chemistry changes, especially under high-energy photons. Accordingly, visible and UV light–induced instability represents another essential degradation pathway warranting detailed discussion.

##### 3.1.4.2 Visible and UV Light

Sunlight, especially its UV component, is another major factor causing perovskite degradation in outdoor conditions, and other conditions are also shown in Figure 8. Although PSCs may remain stable under indoor or white LED light soaking, exposure to full-spectrum sunlight leads to noticeable degradation. This is largely due to the UV-induced deterioration of TiO_2_, commonly used as the ETL in PSCs.^7^ The degradation process in perovskite absorbers under UV and dry air includes:^238,239^

$${\mathrm{3CH}}_{3}{\mathrm{NH}}_{3}^{+}\to {\mathrm{3CH}}_{3}{\mathrm{NH}}_{2}{\mathrm{+ 3H}}^{+}$$

$$I^{‐}{\mathrm{+ I}}_{2}{\mathrm{+ 3H}}^{+}{\mathrm{+ 2e}}^{-}\to \mathrm{3HI}$$

**Figure 8:**
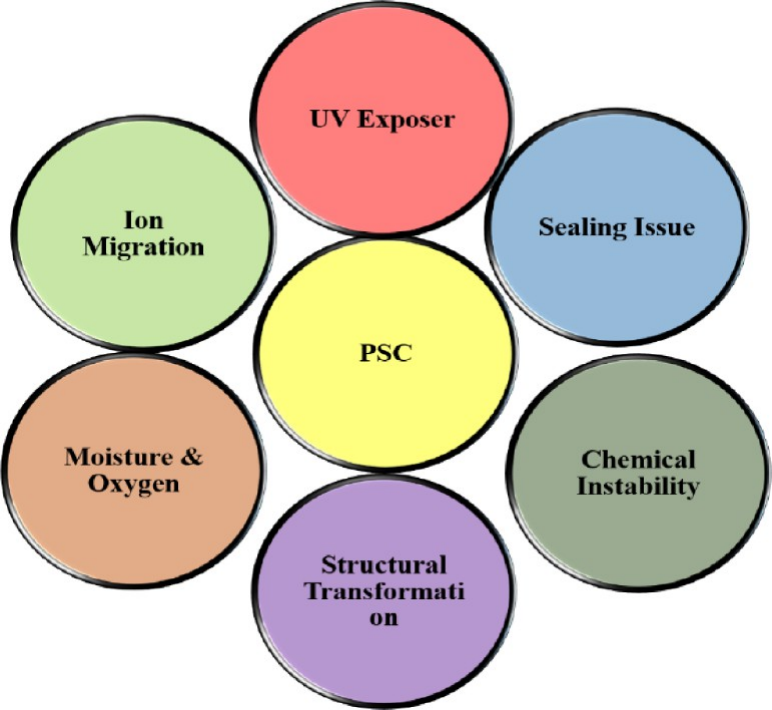
Several instability issues related to the PSCs, created by the author.

In such conditions, the perovskite rapidly breaks down into methylamine, iodine, and lead iodide. One proposed solution is to replace methylammonium (CH_3_NH_3_^+^) with more stable cations, which can reduce degradation and improve device durability.^240^ According to Mei et al., the main reason for MAPbI_3_ perovskite degradation was either the reshaping of the crystal in constrained places or the release of MAI at grain boundaries in an exposed area (Figure 2C). Moreover, the combined effects of electrical bias, heat, and light caused irreversible long-distance ionic migration. The crystalline structure of MAPbI_3_ may be stabilized at the nanoscale by strengthening the grain boundaries with 5-ammoniumvaleric acid (5-AVA) iodide, a bifunctional organic molecule. As a result, the ionic migration became reversible, and the crystal’s dissolution or reshaping was inhibited. This approach offered a reliable means of satisfying the stability criteria for PSCs. Interestingly, a printed PSC implanted with (5AVA)_*x*_MA_1–*x*_ PbI_3_ has proven to be durable, operating for more than 9000 h at a maximum power point of 55 °C ± 5 °C without showing any signs of deterioration. As per the reported articles, light-driven processes often accelerate deeper, intrinsic degradation phenomena, most notably the drift of ionic species and the evolution of metastable defect states. These processes, now widely reported as core contributors to efficiency losses and hysteresis, form the basis of the final subsection addressing ion migration and defect dynamics.

##### 3.1.4.3 Migration of Ions and Other Defects

Perovskite materials are ionic in nature, and their solution-based processing introduces various defects, especially at the grain boundaries and film surfaces. For example, MA^+^ ions may volatilize during annealing, resulting in voids within the film.^241^ Moreover, iodide ions (I^–^) may migrate inside the crystal lattice or toward the electrode interfaces under thermal stress, resulting in electrode degradation and diminished device performance.^242^ Polycrystalline films frequently have irregular grain boundaries, which facilitate moisture infiltration and exacerbate deterioration. Furthermore, rapid and uneven crystal growth can lead to random crystal orientation, hindering efficient vertical charge transport and contributing to lower efficiency and stability.^243^

The following methods have been suggested to improve stability and efficiency in fortin-based perovskite devices: (I) replacing TiO_2_ with alumina; (II) doping TiO_2_ or applying a UV filter to reduce UV-induced photocatalysis; (III) using additives; (IV) replacing hole transport materials; (V) replacing the top electrode; (VI) adding buffer layers; and (VII) producing perovskite material with a different molecular structure.^219^ Yang et al. used a schematic design to illustrate the different types of defects and ion movement in inverted MAPbI_3_ PSCs (Figure 2D­(ai-iv)). MA vacancies (VMA) and I vacancies (VI) are primary Schottky defects, although Pb^2+^ vacancy defects are less common due to large energy barriers for their generation. Ion migration, especially that of MA^+^ and I^–^, is therefore likely to occur at ambient temperature, whereas Pb^2+^ migration needs thermal stimulation. At 85 °C, this migratory process becomes more intense, causing MA^+^ ions to exit and create PbI_2_. Furthermore, Pb ion migration is shown during continuous thermal aging (Figure 2Daiii), and metallic Pb^0^ defects appear on the MAPbI_3_ surface when the I/Pb ratio is decreased (Figure 2Dai). When interstitial ions couple with vacancies, Schottky defects frequently coexist with Frenkel defects (Figure 2Daiv).^65^ Device deterioration results from structural alterations in MAPbI_3_ perovskite materials caused by this migration of ions and defect accumulations at high temperatures.^241^ Furthermore, the volatilization of halide species and the organic cation, especially when MA-containing compounds are present, can cause perovskite films to deteriorate for longer periods of time at lower temperatures.^244^ Therefore, improving the thermal stability of organic and inorganic hybrid perovskites is imperative. The operational stability of perovskite solar cells is often limited by several factors, which can be categorized based on the type of instability, their underlying causes, and the resulting impact on device performance. These aspects are summarized in Table 4.

**Table 4:** Types of Instability in Perovskite Solar Cells, Their Underlying Causes, and the Resulting Consequences on Device Performance

Instability Type	Cause	Consequence
Moisture	Humidity	Hydrolysis of perovskite
Thermal	Heat (>85 °C)	Phase decomposition
UV Light	Sunlight exposure	ROS formation, degradation
Oxygen	Ambient air + light	Superoxide-induced breakdown
Ion Migration	Electric field/heat	Hysteresis, instability
Interface Issues	Poor material compatibility	Interfacial degradation
Phase Transition	Temperature, humidity	Nonphotoactive phase formation

With the key instability pathways established, the next major challenge highlighted across recent studies concerns the transition from laboratory-scale devices to manufacturable modules.

### 3.2 Scalability, Reproducibility, and Film Uniformity

Although small-area PSCs have made great progress, it is still very difficult to achieve the same high efficiency in large-area modules. The shift from spin-coated devices to scalable architectures frequently results in nonuniform film production over extensive substrates, leading to spatial discrepancies in thickness, grain size, and defect density. Heterogeneity among batches and interface inconsistencies are other ongoing issues that hinder reproducibility, especially when processing variables like solvent dynamics, humidity, and precursor concentration are not carefully controlled. A major obstacle arises from the difficulty of spin-coating—the primary laboratory-scale deposition method—with commercial roll-to-roll or slot-die coating processes, which are essential for scaled, continuous manufacturing. Achieving layer coherence, interfacial integrity, and consistent functionality in large-area devices poses a considerable challenge to the production of PSCs. So, researchers have shifted focus toward developing scalable deposition and post-treatment strategies that enable uniform film growth, controlled crystallization, and reproducible device performance across large areas. The following approaches have emerged as key enablers of scalable PSC fabrication.

#### 3.2.1 Scalable Deposition Techniques

The production of perovskites is a crucial step since stability and efficiency are dependent on it. Many attempts have been made to achieve uniformity and high crystallinity in the peroxide films since these factors directly impact the optoelectronic characteristics, dynamics, charge recombination, fusion length, etc., in PSC. The efficiency of PSC is currently comparable to that of crystalline silicon technology. However, the majority of high-efficiency devices (>20%) described in the articles have been manufactured with small areas (>1 cm^2^), and the most popular technique with claimed efficiencies of over 20% is spin coating in an inert atmosphere or glovebox.^245–250^ Research interests are moving toward scalability, or the creation of large-area devices, mini-modules (less than 200 cm^2^), and submodules (more than 800 cm^2^) in an ambient environment, and commercializing perovskite solar cells. The efficiency of the lab-scale devices drops sharply as the active area increases to approximately (1.4 × 10^2^ cm^2^) .^249^ Using inkjet printing, Panasonic is already producing perovskite solar modules with an 802 cm^2^ area and 16% efficiency.^251^ Without a glovebox, Tomulescu et al. reported fabricating PSC to investigate the impact of infrared radiation while creating the perovskite absorber layer with 8% efficiency.^252^ Zakutayev et al. showed 19% efficiency on a 1.96 cm^2^ area of PSC made via blade coating.^250^ PSC can also be made using various techniques, such as screen printing, chemical vapor deposition, electrodeposition, thermal deposition, sequential vacuum deposition, doctor blading, slot-die coating, ultrasonic spray coating, Gravure printing, Screen printing, Inkjet printing, and spray coating, etc.^253–264^ The features of the fabrication methods for perovskite solar cells given in the Table 2. All illustrated in Figure 9. Nonetheless, spin coating in small-area devices and blade coating, spray coating, screen printing, and coating yield encouraging outcomes.

**Figure 9:**
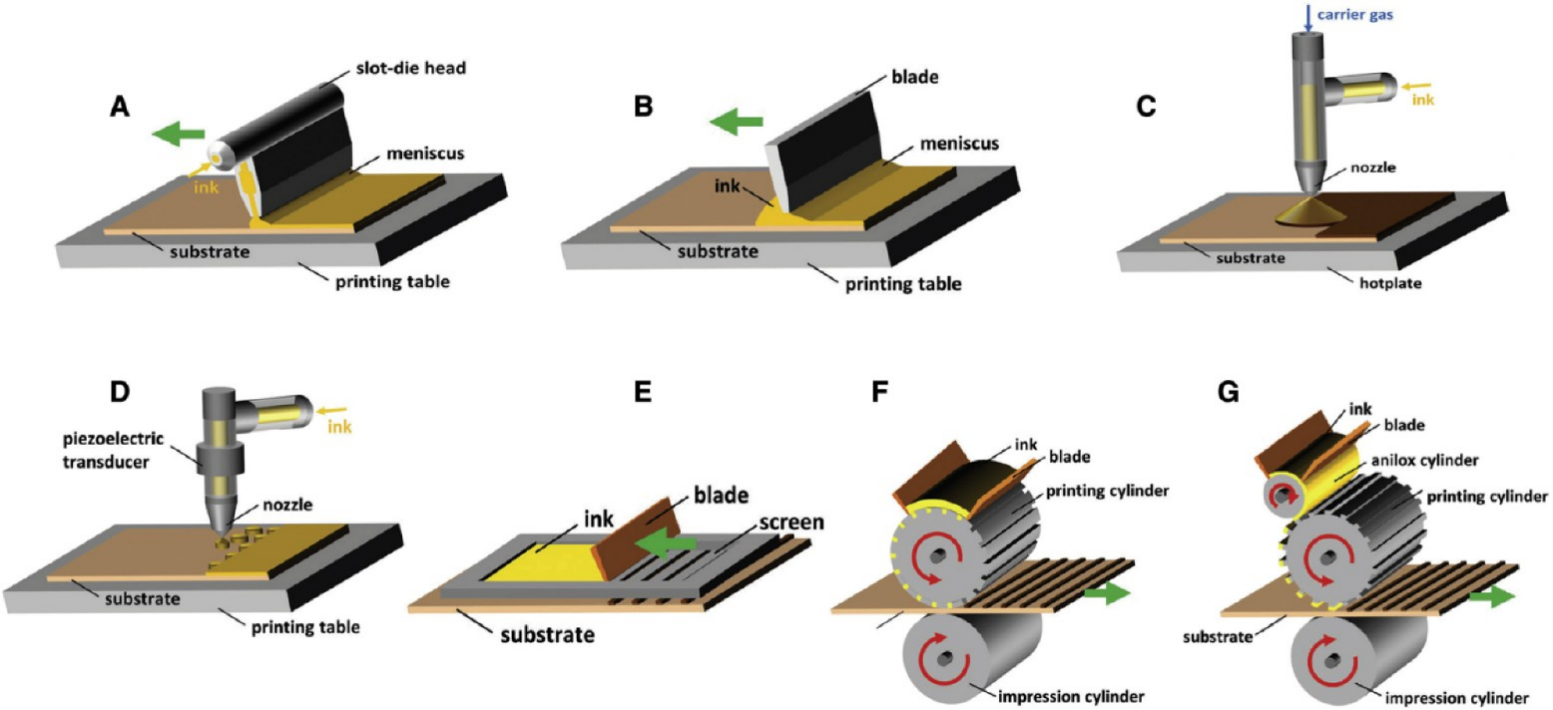
Pressure-Assisted Lamination (PAL) scale-up coating methods (A) Slot-die coating. (B) Blade coating. (C) Spray coating. (D) Inkjet printing. (E) Screen printing. (F) Gravure printing. (G) Relief printing. Reuse with permission, Copyright,[Bibr ref265] 2016.

#### 3.2.2 Spin Coating

Among the various fabrication techniques developed for perovskite solar cells (PSCs), two of the most commonly used methods are the one-step coating and the two-step sequential coating processes. In the one-step coating technique, all the necessary precursor chemicals such as lead iodide (PbI_2_), methylammonium iodide (MAI), and formamidinium iodide (FAI) are dissolved together in a common organic solvent, typically dimethylformamide (DMF) or dimethyl sulfoxide (DMSO). However, this method is more suitable for small-area device fabrication and is not ideal for large-scale production due to issues such as material wastage and poor film uniformity over larger areas. The two-step sequential coating technique offers good control over film formation and device reproducibility. This technique was first demonstrated by Chen et al.,^263^ where PbI_2_ is initially deposited on a mesoporous TiO_2_ scaffold using spin coating and then annealed at a low temperature (around 70 °C). Subsequently, the film is dipped in a methylammonium iodide solution in isopropanol, which allows for the intercalation of the organic cation and the formation of a complete perovskite film. Often achieving PCS above 15%, this method produces improved film uniformity and higher consistency in device performance. Nonetheless, similar to the one-step technique, the two-step procedure also incurs significant precursor wastage during spin coating and is inappropriate for direct scaling to perovskite modules.

Furthermore, advanced methodologies and doping strategies have been investigated to address scalability and device stability issues. An improved production technique employing heavily doped inorganic oxide layers to serve as charging layers in multilayer perovskite solar cells was reported by Grätzel et al. in 2015. P-doped (p^+^) Ni_*x*_Mg_1–*x*_O was employed as the HTL, whereas n-doped (n^+^) TiOx functioned as the ETL. Doping was achieved by substituting Ni^2+^ or Mg^2+^ ions in the Ni_x_Mg_1‑x_O lattice with Li^+^, and replacing Ti^4+^ ions in TiO_*x*_ with Nb^5+^. This approach enabled the deposition of exceedingly thin oxide layers (10–20 nm), significantly reducing series resistance while improving shunt resistance. The doped layers enhanced charge transport properties and reduced recombination, resulting in a device effectiveness of 15% for an active area exceeding 1 cm^2^. In 2016, the same group introduced a vacuum-assisted solution method (VASP) for large-area, high-efficiency perovskite solar cells (PSCs).^266^ In this process, a precursor solution with a composition of FA_0.81_MA_0.15_PbI_2.51_Br_0.45_ dissolved in DMSO was spin-coated on top of a mesoporous TiO_2_ layer. The wet film was immediately transferred into a low-pressure (20 Pa) vacuum chamber for a few seconds. This step accelerated solvent evaporation, leading to the formation of an orange-colored intermediate perovskite film, which was then annealed at 100 °C for 30 min. The result was a shiny, smooth, and highly crystalline perovskite layer with large grain sizes (400–1000 nm) and no pinholes. Upon deposition of a spiro-OMeTAD HTL and a thermally evaporated gold layer, the final device achieved an efficiency of 19.5% over a substrate area greater than 1 cm^2^. Further improvements have included the use of Cl-capped TiO_2_ nanoparticles as the ETL to reduce charge recombination at the perovskite/ETL interface, which also led to a high efficiency of 19.5% on a 1.1 cm^2^ device.^267^ Similarly, to get careful control of the residual PbI_2_ content within the perovskite layer has helped achieve PCEs as high as 20.1% in 1 cm^2^ devices.^268^ Although these advances such as spin coating, remains limited for module-scale fabrication due to issues such as uneven film thickness over large areas. So, the rate-limiting factor in perovskite crystallization during scale-up, some limitations pointed toward the need for more scalable and environmentally benign deposition techniques for the commercialization of PSCs.^269–271^ To overcome these challenges, researchers have introduced a variety of scalable deposition and quality-control strategies, discussed in detail below. Also, a summary of these major fabrication methods, including Feature, One-Step Coating, Two-Step Sequential Coating, Vacuum-Assisted Solution Process (VASP), and Doped Inorganic Layers, is provided in Table 5.

**Table 5:** Fabrication Methods for Perovskite Solar Cells

Feature	One-Step Coating	Two-Step Sequential Coating	Vacuum-Assisted Solution Process (VASP)	Doped Inorganic Layers
Process steps	Single-step precursor deposition	PbI_2_ coating followed by MAI dipping	Spin coating + vacuum drying + annealing	Doping of HTL/ETL with metal ions
Film uniformity	Moderate	Good	Excellent	Excellent
Scalability	Poor	Limited	Improved (more scalable)	Good (suitable for >1 cm^2^ devices)
Material wastage	High (via spin coating)	High	Moderate	Low
Device efficiency achieved	∼15–18%	∼15–20.1%	Up to 19.5% (certified)	15% (with doped layers)
Grain size	Controlled growth	Improved crystallinity	Large grains (400–1000 nm)	Not grain-related (focused on charge layers)
Key drawbacks	Precursor waste, solvent use	Not scalable for modules	Equipment requirement, more process steps	Doping complexity, material cost
Advantage	Simpler fabrication	High reproducibility	High-quality films, reduced defects	Better charge extraction, improved conductivity

#### 3.2.3 Scalable Methods

##### 3.2.3.1 Blade Coating

Blade coating is an easy and cost-effective method used to get large-area thin films, and it works well for roll-to-roll production. In this technique, a liquid solution is poured onto a flat surface and spread evenly by a horizontal blade. As the solvent slowly evaporates, a thin film of material forms. Since the solvent dries naturally and not quickly like in spin coating, it produces larger films, but sometimes the films are less dense. The thickness of the film depends on several factors, like the solution concentration, its thickness, the gap between the blade and the surface, and the speed of the blade. Yang et al. conducted a study indicating that heating the surface to approximately 100 °C prevents undesirable intermediate phases, resulting in an efficiency of 13–14%.^272^ Upon raising the temperature to 150 °C, the efficiency enhanced to 17%. Humidity plays a substantial role in the development of these coatings. Deng and his team found that sustaining humidity levels between 15% and 25% promoted the creation of high-quality perovskite crystals, achieving an effectiveness of 10.44% even in open air.^273^ Zeng and colleagues developed a rapid blade coating method that enabled quick and efficient film formation using a low amount of the surfactants L-α-phosphatidylcholine.^274^ This method attained performances of 15.3% and 14.6% for modules with areas of 33 cm^2^ and 57.2 cm^2^, respectively. Bade et al. investigated the influence of various surfaces on film development in humid environments. The utilization of a specialized self-assembled monolayer (SAM) enhanced film characteristics and device performance, achieving 18.47% efficiency in tiny devices and 14.64% in larger ones. Their devices also showed good long-term stability and even worked well in perovskite mini-modules with 14.13% efficiency on an 18 cm^2^ area.^275^

##### 3.2.3.2 Slot-Die Coating

Slot-die coating is quite similar to blade coating. This coating has a more controlled method to get a uniform thin film. The solution is pumped through a narrow slot and deposited on a moving surface. The thickness of the film depends on how fast the solution is pumped, it can lead to problems like pinholes or uneven crystal growth. Some researchers reported this problem and their solution. In 2017, Baker et al. made almost an entire solar cell using only slot-die coating, except for the metal parts.^276^ They used an external gas quenching method via blowing nitrogen gas to speed up drying. This helped them achieve 11.96% efficiency for small devices. Qin’s team later showed that using preheated surfaces and blowing cold air improved film smoothness, achieving 9.2% efficiency.^277^ Cheng and colleagues used a new HTL material called bifluo-OMeTAD, which helped prevent crystallization problems and improved efficiency to 14.7%.^278^ Di Giacomo et al. worked on reducing hysteresis effects by using potassium as a passivating layer and got 17.18% efficiency on small devices and 15% on flexible modules (5 × 6 cm^2^).^279^ Fievez et al. made large modules (12.5 × 13.5 cm^2^) using slot-die coating and laser patterning, reaching 10% efficiency.^280^ Another group created mesoscopic solar cells with an efficiency of 12.87% on a 60.08 cm^2^ active area. Gittens recently achieved over 18% efficiency using gas and heat-assisted crystallization with a special formula of CsFA-based perovskite.^281^ They also managed to get over 11% efficiency on 10 × 10 cm^2^ modules without adding chemicals, showing how slot-die coating can be used for large-scale production.

##### 3.2.3.3 Spray Coating

Spray coating is one of the fastest and scalable methods used in industries. It works by spraying very tiny droplets of a solution onto a surface. These droplets come together to form a wet layer to get uniform and thin film. then dries to form a thin film. The quality of the film depends on the size of the droplets, the type of spray nozzle, the temperature of the surface, and the distance between the nozzle and the surface.^282,283^ If the nozzle is too close, it causes holes; if it is too far, powder forms. Heo’s group showed that spraying a CsPbI_3_ solution onto a CsPbI_2_Br film creates a graded perovskite structure. This improved how long charge carriers live and widened the light absorption range, giving a 16.81% efficiency for a small device. Researchers also made larger modules with 13.82% efficiency (112 cm^2^) that retained most of their performance even after 1000 h under light.^284^ Das and team tested different conditions and solvents and achieved 11% efficiency.^285^ Chou et al. made perovskite solar cells on glass (13%) and on flexible plastic (8.1%), using a photonic curing method to improve TiO_2_ layers.^286^ Two-step spray coating surpasses one-step application by producing more homogeneous coatings with enhanced stability and improved charge flow. Liu et al. noted that early precipitation led to nonuniform films.^287^ Chou et al. developed 10 × 10 cm^2^ modules exhibiting 15.5% efficiency through the alteration of solvent ratios to yield larger grains.^288^ The utilization of a megasonic nebulizer functioning at a frequency of 1.7 MHz recently produced finer raindrops and larger particles, with a success rate of 16.4% on 1 cm^2^ devices. Park’s team created a completely spray-coated inverted solar cell that attained an efficiency of 10.09%, illustrating the scalability of this method.^289^

### 3.3 Other Novel Scalable Techniques

Some studies utilize a different method to achieve enhanced PSCs. Zhang et al. developed an innovative precursor solution intended for the deposition of perovskite films on large, flexible, or stiff substrates.^290^ The literature research indicates that spin coating is suitable for small areas; however, it is impractical for large-scale production due to the extended drying time and solvent-related film defects. To resolve this, Chen et al. utilized a different way. A resilient MAPbI_3_ sample was placed in a hermetically sealed room filled with methylammonium (MA) vapor.^291^ Liu et al. developed an innovative method that excluded the use of solvents or vacuum.^292^ They amalgamated specific precursor powders and placed the resultant mixture onto a substrate, eventually overlaying it with a thin film. They then employed pneumatic pressure to evenly spread the mixture and raised the temperature to 50 °C. This produced a homogeneous and dense perovskite layer distinguished by significant granules. They achieved 12.1% efficiency on a significant 36.1 cm^2^ solar panel.

### 3.4 Vapor-Assisted Deposition (VAD) of Perovskites

The vapor-assisted deposition technique combines the precision of solution processes with the benefits of vapor-phase methods. The process comprises two phases: first, a precursor layer, such as lead iodide (PbI_2_), is spin- coated onto a substrate. Second, the film is exposed to organic halide vapors, such as methylammonium iodide (MAI) or formamidinium iodide (FAI), under controlled temperature settings. This exposure facilitates a gas–solid process that transforms the precursor substance into a highly crystallized perovskite structure. VAD fabrications offer controlled reaction kinetics, resulting in dense, pinhole-free films with large grains, reduced trap states, and improved long-term environmental stability. Chen et al. (2013) elucidated a sequential deposition method utilizing MAI vapor to convert an existing PbI_2_ layer into CH_3_NH_3_PbI_3_ perovskite. This method led to a notable improvement in film shape and photovoltaic efficiency, achieving a PCE of 15%.^263,293^ Subsequently, Saidaminov et al. (2015) developed a vapor-assisted solution process (VASP) for the manufacture of planar heterojunction perovskite solar cells. Their methods produced uniform and homogeneous films with minimized grain boundaries and improved coverage, leading to enhanced charge transport and increased device stability.^294^ Some researchers have demonstrated that this methodology provides superior benefits relative to other established strategies. Saidaminov et al. (2015) employed a vapor-assisted crystallization technique to produce single-crystalline MAPbI_3_ perovskite films. The approach enabled the control of crystal size, thickness, and orientation, yielding materials with carrier diffusion lengths exceeding 175 μm and significantly enhanced photoluminescence lifetimes. Their research was essential in demonstrating the scalability and fault tolerance of VAD-grown single crystals for optoelectronic applications.^295^ A straightforward solution-based technique was employed to deposit PbI_2_, subsequently processed using hybrid chemical vapor deposition. This approach demonstrated notable efficiency. Figure 10A­(a) illustrates the HCVD process, utilizing lead chloride (PbCl_2_) and methylammonium iodide (MAI) as the initial ingredients.^296^ The substrates, composed of fluorine-doped tin oxide (FTO) glass with a TiO_2_ layer and predeposited PbCl_2_, are situated in one temperature-regulated section of the furnace, whereas the MAI is positioned in a distinct section. The furnace is then sealed, the air is removed to create a vacuum, and an inert gas is passed through. The MAI moves in two steps: first, it travels in the gas phase to reach the substrate (gas-phase diffusion), which depends on temperature and pressure (Dg ∝ T^3/2^/P). After reaching the substrate, MAI must move through the film to react with the PbCl_2_ at the bottom (solid-state diffusion). This step is controlled by the Arrhenius equation (Ds ∝ e^–^_^C/*kT*^_), where Ds is the solid diffusion rate, C is a constant, *k* is Boltzmann’s constant, and *T* is temperature.^297,298^ Raising the temperature increases both gas and solid diffusion, which helps speed up the formation of the perovskite layer. Figure 10A­(b) shows a diagram of the full solar cell structure and all its layers.

**Figure 10:**
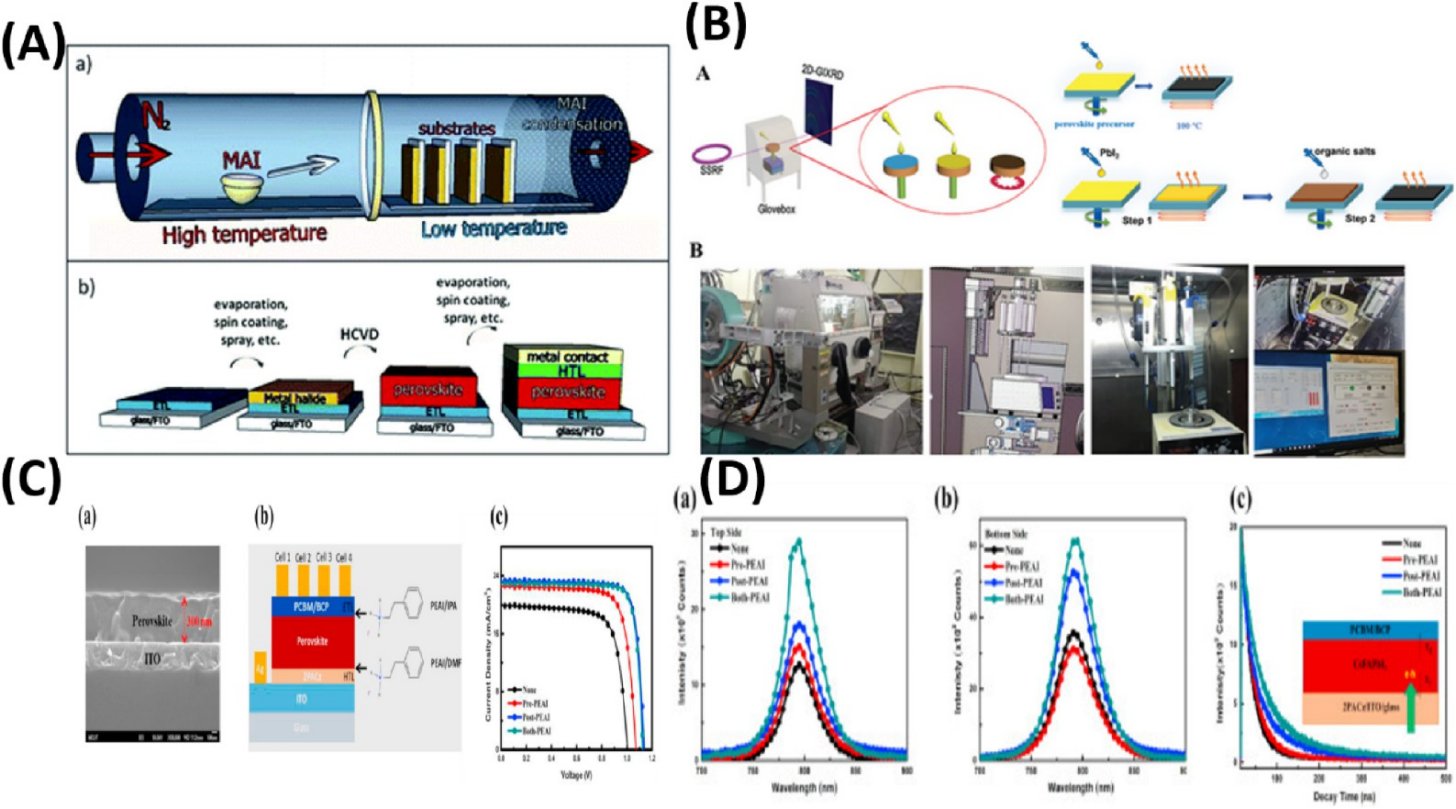
(A) HCVD-based perovskite fabrication a- diagram of the HCVD furnace and MAI deposition onto metal halide seeded substrates. b- A full solar cell’s layered structure made using the HCVD technique. A glass substrate, an FTO layer, an electron transport layer, a perovskite layer, a hole transport layer, and a top metal contact make up a whole solar cell. Reproduced with permission, Copyright,[Bibr ref299] 2016. (B) a- Synchrotron radiation facilities’ in situ GIWAXS systems for solution spin-coating. b- The setup at SSRF, where the preparation conditions are in a typical glovebox of N_2_ to prevent the effects of oxygen and ambient moisture (c (H_2_O, O_2_) < 1 ppm). Reproduced with permission, Copyright, [Bibr ref298],[Bibr ref299] 2024, 2016. (C) a- Perovskite cross-sectional scanning electron microscopy picture; b- Device architecture; c- PSCs’ J-V curves under a single sun’s light; and (D) PL spectra of the samples under various treatments. a-Top excitation, b- bottom excitation, and c- sample TRPL spectra. Reproduced with permission, Copyright,[Bibr ref305] 2019.

Shao et al. employed synchrotron radiation-based in situ grazing-incidence wide-angle X-ray scattering (GIWAXS) techniques to report perovskite-based devices by resolving the real-time crystallization paths of perovskites during the spin-coating process. high resolution. This allows for the bridge of complex perovskite structure information with device performance.^299^ The basic configuration and experimental flow of the line station layout and the in situ glovebox device are depicted in Figure 10B. Specifically, the optical shack is used to introduce the quasi-monochromatic X-ray beamline of synchrotron radiation into the experimental shed, the beryllium window on the left side of the glovebox illuminates the homogenizer platform inside the glovebox, and a 2D detector (CCD or Pilatus) collects the diffraction or scattering signal. The experimental setup for the spin-coating study employing in situ GIWAXS technology based on the SSRF is displayed in first part of Figure 10B. It is possible to implement the one-step and two-step spin-coating processes independently. An N_2_-filled glovebox with a spin-coater integrated into the beamline is used to deploy an in situ GIWAXS experimental setup at the SSRF’s beamline stations, as seen in other part of Figure 10 B.

More recently, VAD has been optimized for multiplication and mixed halide perovskite systems, enabling fine-tuning of optoelectronic properties, thermal stability, and bandgap engineering for Tandem Solar Cells (TSCs) and light-emitting devices. Researchers have focused on ambient-air compatible VAD processes, compositional engineering of the vapor phase, and tuning of substrate temperatures and exposure durations to control the crystallization pathway, phase purity, and grain orientation.

### 3.5 Process Monitoring and In Situ Characterization of VAD-Grown Perovskites: GIWAXS and PL Microscopy

To understand and control the film formation process during VAD, in situ characterization techniques are critical. Two of the most informative methods used are grazing incidence wide-angle X-ray scattering (GIWAXS) and photoluminescence (PL) microscopy, which provide complementary information about structural and optoelectronic evolution during film development. GIWAXS method is sensitive to surface and near-surface structures. So, it makes it ideal for observing thin-film perovskite nucleation, phase transformation, grain orientation, and growth kinetics. In 2019, Guo et al. used in situ GIWAXS to investigate the intermediate phases during the MAI–PbI_2_ conversion in VAD.^300^ According to their findings, after extended exposure to vapor, metastable hydrate-like layered phases changed into the final perovskite structure. These characteristics offer the advantage of regulating reaction environments and humidity for consistent crystallization. Stoumpos et al. (2013)^301^ additionally investigated the influence of temperatures and vapor content on perovskite crystallization by in situ GIWAXS. Their findings suggested that altering the temperature limit could determine the preferred orientation of grains, influence the pace of crystallization, and impact the final grain size. They concluded that extended vapor exposure at ideal temperatures resulted in better organized structures and reduced microstrain, hence improving electronic properties. GIWAXS observes structural changes, while in situ PL microscopy provides real-time insights into optoelectronic properties during crystallization. The intensity, duration, and spectral variations of this approach signify trap density, defect passivation, and carrier recombination dynamics. PL is an essential complement to structural probes like GIWAXS.

A relationship between photoluminescence behavior, lattice dynamics, and defect tolerance in hybrid perovskites was established by Brivio et al. in 2015 ^302^ and by Ni and Gao in 2020.^303^ Their static photoluminescence studies revealed that increased photoluminescence intensity correlated with improved crystallinity and a decrease in nonradiative recombination centers.^302,303^ In 2020, Gao et al. combined in situ GIWAXS and PL microscopy to elucidate perovskite crystallization during VAD [303] . They concurrently monitored diffraction peaks and the increase of photoluminescence (PL) signals, revealing that the maximum PL intensity corresponded with the dissipation of precursor phases and the formation of well-structured perovskite domains. Their research provided critical insights for improving precursor exposure duration and annealing parameters, showing a direct relationship between structural ordering and optoelectronic performance.^304^ Utilizing these in situ devices, researchers can now precisely monitor and modify the developmental dynamics of perovskite films during VAD. These insights facilitate defect mitigation, enhanced phase regulation, and performance improvement in diverse perovskite optoelectronic devices. Zhong et al. employed a PbI_2_-rich inverted architecture comprising ITO/2-(9H carbazol-9-yl)­ethyl)­phosphonic acid (2PACz)/perovskite (300 nm), as illustrated in Figure 10C­(a), together with PC61BM/BCP/Ag, depicted in Figure 10C­(b), utilizing Cs_0.18_FA_0.82_PbI_3_ perovskite. The study examined the effects of a PEAI-based post-treatment approach on the perovskite layer and the consequences of pretreating the hydrophobic 2PACz substrate with PEAI. Figure 10D displays the PL spectra of the ITO/2PACz/perovskite/PCBM/BCP samples under both top and bottom excitation. The presence of asymmetric PL peaks indicates two distinct emission pathways.^305^ Notwithstanding considerable advancements in scalable deposition and in situ diagnostics, PSC technology encounters substantial challenges that impede commercial viability. Environmental degradation, ion migration, and batch-to-batch variability persistently restrict device longevity and repeatability. Furthermore, issues concerning lead poisoning and industrial standardization continue to hinder wider implementation.

#### 3.5.1 Module Engineering and Encapsulation Strategies

Maintaining constant electrical and optical performance in hybrid perovskite solar cells from small lab-scale devices to large-area modules presents significant challenges. The enlargement of these PSCs results in issues such as heightened resistance, current disparity, edge shunting, and water vapor infiltration, all of which diminish power conversion efficiency and device stability. Thus, strong module design and encapsulation are essential to protect the perovskite layer from environmental deterioration and to maintain uniform electrical output across interconnected cells. Advanced approaches, including laser scribing for precise connections and the optimization of layer interfaces, diminish resistance and optical losses while enhancing scalability. This happens only utilizing encapsulation of the materials, including UV-curable epoxy resins, multilayer polymers, and self-healing coatings, which obstruct oxygen, moisture, and other detrimental elements. The PSCs must have a great encapsulation to remove the extrinsic instability. Other types of solar cells frequently employ the encapsulation process, which may protect the device from oxygen and moisture exposure while enhancing thermal and mechanical stability to function in unpredictable weather. To investigate the internal stability of PSC, fine encapsulation also aids in avoiding external influences.^306,307^

Thin film encapsulation (TFE), which involves depositing a thin film as an encapsulant on top of the module (Figure 11A­(a)), and edge seal, which involves applying sealant around the module (Figure 11A­(b)), are the two general techniques into which conventional encapsulation systems may be separated. Han et al. used three hot melt films—polyurethane, polyolefin, and EVA—to encapsulate printable PSCs via screen printing (Figure 11Ba).^308^ They found that temperatures up to 80 °C were safe, but higher temperatures, like 100 °C and 120 °C reduced *V*_OC_. So, encapsulation should be done below 100 °C. The PCE loss was due to interface changes, not perovskite damage. Large modules (10 cm × 10 cm) encapsulated with PU and sealed with silicone stayed waterproof for over 30 days, retaining 97.52% of the original PCE after 2136 h outdoors. Shi et al. included a PMMA/rGO (PRC) layer,^309^ enhancing moisture and thermal resistance. In the absence of rGO, scientists found that after 1000 h at 35 °C and 40% relative humidity, over 90% power conversion efficiency was maintained after water immersion with no change. Similarly, Choi et al. examined the pressing force in lamination using polyolefin (Figure 11 Bb).^310^ A pressure of 400–500 mbar enhanced performance by reducing resistance and hysteresis. The Time-Resolved Photoluminescence (TRPL) and impedance results confirmed improved charge transmission. This pressure also increased PCE by almost 7% compared to the average. Lamination is widely employed in silicon solar cells and serves as a feasible method for perovskite solar cells (PSCs). Optimal temperature and pressure are essential; excessive heat compromises perovskite, while inadequate heat prevents the layer from melting. Optimal pressure can augment efficiency. An issue is mitigating the damage inflicted by insufficient energy input during atomic layer deposition (ALD). A 50 nm Al_2_O_3_ layer was deposited by Li et al. using ALD (Figure 11C (a)).^311^ Similar to this, Li et al. employed plasma-enhanced atomic layer deposition (PEALD) at 50 °C after first depositing ethylene glycol as a buffer layer using molecular layer deposition (MLD) (Figure 11C).^312^ Lead sequestration strategies often rely on chemical chelators or functional barrier materials that actively bind to Pb^2+^ ions and prevent their release. Pb-chelating agents, such as crown ethers, EDTA-functionalized layers, or phosphonic acid-based polymers, have shown high Pb-binding affinity and are integrated either within the encapsulation layer or coated as surface treatments on PSCs.^313^ In some designs, ion-exchange resins and metal–organic frameworks (MOFs) have also been explored for their selective and high-capacity Pb adsorption properties. These strategies aim to localize and neutralize lead in case of accidental damage to solar panels, by reducing environmental exposure risks using some methods. To resolve the obstacle, some methods, such as the integration of encapsulation and Pb-sequestration methods are good for making scalable device architectures. This technique not only improves lead-based PSCs but also moves the technology closer to environmentally responsible commercialization. Recent reviews have emphasized the importance of combining both physical barriers and chemical mitigation for comprehensive lead containment.^313^ As commercialization progresses, such hybrid mitigation approaches will play an essential role in addressing environmental sustainability concerns associated with high-efficiency Pb-based perovskite photovoltaics.

**Figure 11:**
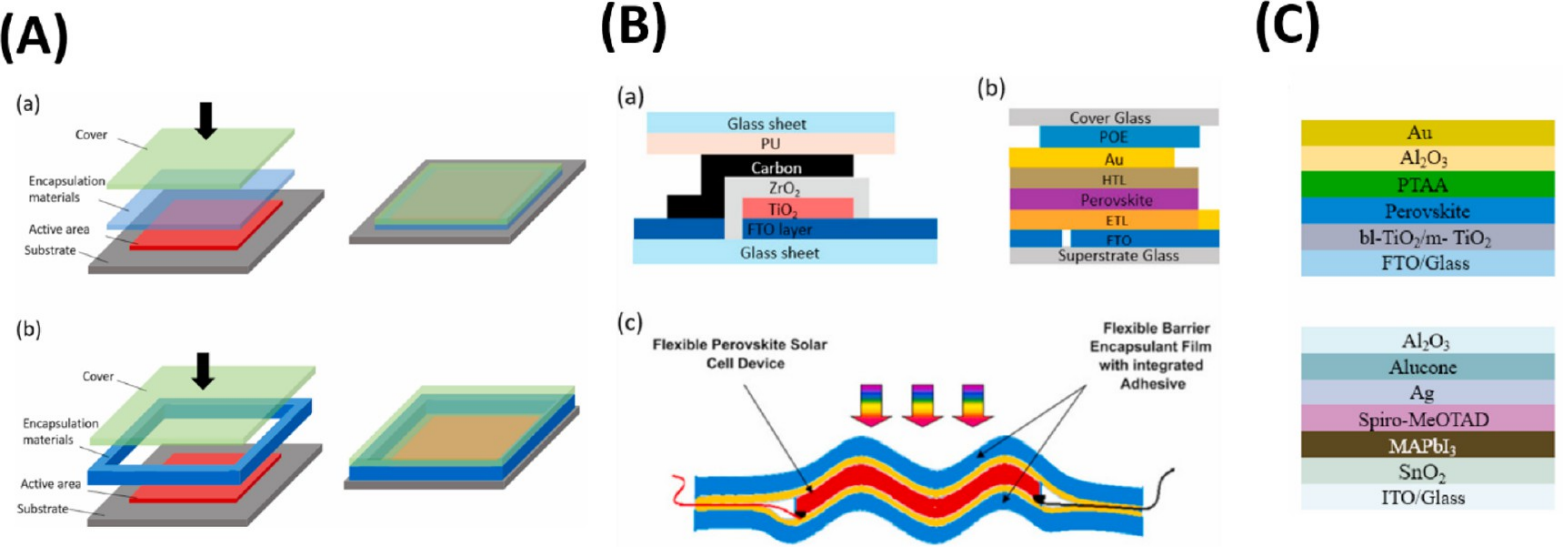
(A) a-b- The protective layer covering the active region is made of encapsulating materials. The active area’s edge is where the sealant is applied. Reproduced with permission, Copyright,[Bibr ref316] 2020. (B) a- PSCs were encased in PU as TFE, and the rear cover was made of a sheet of glass.^306^ b- Polyolefin as a TFE-encapsulated PSC contains a glass/FTO/c-TiO_2_/mp-TiO_2_/perovskite/Spiro-OMeTAD/gold structure. Reproduced with permission, Copyright,[Bibr ref314] 2022. c- TFE as a flexible barrier with integrated adhesive. Reproduced with permission, Copyright,[Bibr ref315] 2021. C- First After being encapsulated by Al_2_O_3_, PTAA-based PSCs exhibit improved thermal stability. Reproduced with permission, Copyright,[Bibr ref311] 2024. Second, the devices are protected from PEALD by the low-damage alucone, which acts as a buffer layer. Reproduced with permission, Copyright,[Bibr ref312] 2025.

### 3.6 Standardization and Protocol Harmonization

A major challenge in moving hybrid perovskite solar cells (PSCs) from lab success to industry is the lack of standardized testing and fabrication methods. Although efficiencies over 25% have been achieved, inconsistent testing conditions, reporting standards, and limited long-term stability data make it hard to compare results and slow commercialization. Many other devices are tested in ideal lab setups without certification or durability checks, leading to issues when scaling. So, the PV is adopting global frameworks like the International Summit on Organic Photovoltaic Stability (ISOS) and IEC standards that set clear rules for reliability and environmental testing. Protocols such as ISOS-L (light soaking), ISOS-T (thermal), and ISOS-D (dark storage) help measure device lifespans realistically. Applying these during research and pilot production improves transparent and reproducible reporting. Standardizing the fabrication process, including solvent use, humidity control, annealing, and layer thickness. As PSCs scale to large modules, durability tests like damp heat, UV exposure, and thermal cycling will ensure they withstand real-world conditions. Certification schemes modeled after silicon solar panels will confirm PSC modules maintain performance under stress. Life-cycle assessment and lead safety checks will further align PSCs with sustainability goals. Overall, consistent testing and manufacturing processes will help ensure academic advances are genuine and reproducible, speeding PSCs’ industrial and market entry.^317^

### 3.7 Sustainability and Commercialization Prospects of Hybrid Perovskite Solar Cells

#### 3.7.1 Environmental Issues of Lead

PSC lead leakage is a serious risk to the environment and human health, especially when devices are damaged or exposed. Even though lead-free perovskites are being developed, lead-containing perovskite solar cells are still widely used due to their lower stability and efficiency. Perovskite solar cells (PSCs) degrade into lead iodide (PbI_2_) when subjected to light, heat, or humidity. PbI_2_ is soluble in water, and precipitation or humidity may deteriorate parts or the entire perovskite layer if the device is affected. Despite the small lead concentration in a single PSC according to safety regulations, the aggregation of multiple devices in a confined area may lead to lead accumulation in soil and vegetation, consequently invading the food chain. Lead exposure is harmful to humans and the environment because it disrupts important biological processes like digestion, blood cell formation, and the neurological system; children are particularly susceptible to this effect.^318–330^ Perovskite solar cells (PSCs) leak lead into the environment, which can harm health and the environment in the long run. This is especially true in areas where there are many installations. Researchers are currently focused on finding ways to reduce lead leakage and recycle lead from broken or abandoned equipment in order to solve these concerns. Methods for safely recovering and reusing lead, enhanced encapsulation, and materials that can absorb lead leakage are all part of the current strategy. More people should be aware of the need to prevent lead toxicity, and these methods can help reduce pollution and health hazards.^318–330^ Also, perovskite solar cells offer a variety of advantages and face specific challenges depending on the material system employed. A comparative overview of their key merits and demerits is summarized in Table 6.

**Table 6:** Key Merits and Demerits of Perovskite Solar Cells

S.NO.	Merits	Demerits
**1**	**Hybrid halide-based perovskites:** hybrid halide-based perovskites with the formula ABX_3_, where A is for organic or inorganic cation, B is for divalent metal such as Pb^2+^, Sn^2+^, or Ge^2+^, and X is for halide ion such as I^–^, Br^–^, or Cl^–^, offer tunable optoelectronic properties and wide bandgap versatility.	**Environmental instability:** PSCs and their films are highly sensitive to external factors such as moisture, heat, oxygen, and UV light, leading to degradation of the active layer and loss of performance.
**2**	**High efficiency:** perovskite solar cells have achieved >26% PCE owing to high absorption coefficients, long carrier diffusion lengths, and defect-tolerant electronic structures.	**Water sensitivity:** exposure to humidity leads to hydrolysis of the perovskite lattice, causing irreversible decomposition into PbI_2_ and halide species.
**3**	**Low-cost and solution-processable:** simple fabrication techniques (spin-coating, blade coating, slot-die coating) enable large-area and flexible device manufacturing with low energy input.	**Thermal instability:** lead halide perovskites exhibit poor thermal resilience; at high temperatures, decomposition generates volatile halogen gases and metallic lead residues.
**4**	**Lightweight and flexible:** thin-film form factors allow integration into flexible, portable, and building-integrated photovoltaic (BIPV) applications.	**Photo-oxidative degradation:** prolonged exposure to light and oxygen accelerates halide ion migration and organic cation decomposition.
**5**	**Low energy loss and high open-circuit voltage:** minimal nonradiative recombination enhances conversion efficiency compared with traditional silicon-based devices.	**Scalability challenges:** achieving uniform film quality and efficiency over large areas remains difficult, hindering industrial-scale module production.
**6**	**Tunable composition:** adjustable halide and cation combinations enable color-controlled and tandem-compatible solar devices.	**Lead toxicity:** environmental and health concerns arise from potential Pb leakage during fabrication, operation, or disposal stages.

#### 3.7.2 Preventing Lead Leakage

When subjected to heat, moisture, or physical damage over time, lead leakage from perovskite solar cells (PSCs) becomes an extremely pressing matter for both the environment and human health. Chemical encapsulation has emerged as a key strategy for dealing with this problem. Materials used for encapsulation are engineered to bind Pb^2+^ ions strongly by the use of functional groups, including phosphate, carboxyl, and sulfonic acid. These materials significantly lessen the potential for lead to leak into the environment when paired with protective coatings such as aerogels, ethylene-vinyl acetate, or UV-curable polymers. Research has demonstrated that phosphate-based encapsulants are capable of reducing lead emission to below 0.2 ppm, even after being exposed to water for an extended period of time.^331–334^ Alongside external encapsulation, internal alterations to the perovskite layer are also advantageous. Techniques such as 2*D*/3D perovskite stacking, the inclusion of substances that are hydrophobic with fluorinated or extended alkyl groups, and the complexation of Pb^2+^ ions with strong chelators like 1,2-ethanedithiol (EDT) reduce lead leakage. In-situ polymerization at grain boundaries enhances hydrophobic coverage and increases lead-binding sites, hence reducing Pb^2+^ dissolution. The rapid production of insoluble lead compounds, such as phosphates, before water exposure effectively sequesters lead.^100,338^ The integration of internal and external approaches results in lead sequestration efficiencies of 99.9%, hence reducing environmental hazards while preserving PSC performance.

#### 3.7.3 Lead-Free Materials

Lead-based PSCs are more detrimental to the environment. So, other material substituting them with lead-free materials for the fabrication of hybrid PSs with improved PCE.^102,227^ Researchers are consequently developing perovskite materials that integrate high efficiency, long-term stability, and environmental safety. Methylammonium (MA^+^) at the A-site is being substituted with more stable and thermally resilient cations, such formamidinium (FA^+^), cesium (Cs^+^), or their composite mixtures. Alternative metals for the B-site, including tin (Sn^2+^), germanium (Ge^2+^), silver (Ag^+^), bismuth (Bi^3+^), and antimony (Sb^3+^), have been investigated to reduce toxicity and enhance stability. Cesium-based perovskites have demonstrated encouraging outcomes, with CsPbI_3_ and CsPbI_2_Br attaining efficiencies of approximately 19% and 16.7%, respectively, despite the presence of hazardous lead.^103,104^ To overcome this, tin-based perovskites have been proposed as lead-free and environmentally friendly alternatives. Commonly reported tin halide perovskites include CsSnI_3_, CsSnI_2_Br, CsSnIBr_2_, and CsSnBr_3_, but their performance is limited by the rapid oxidation of Sn^2+^ → Sn^4+^, which deteriorates the film quality and device stability. In 2022, Wang et al.^197^ mitigated this issue by adding SnF_2_ to CsSn-based perovskites and reported efficiencies of 1.66% (CsSnI_3_), 1.67% (CsSnI_2_Br), 1.56% (CsSnIBr_2_), and 0.95% (CsSnBr_3_). Later, Wenzhe et al.^105^ demonstrated that incorporating hypophosphorous acid (H_3_PO_2_) effectively reduced Sn vacancies and inhibited Sn^2+^ oxidation, improving CsSnIBr_2_ PSC efficiency by approximately 3%. More recently, Karna et al.^340^ used the SCAPS-1D simulation tool to optimize defect and charge transport layers in CsSnIBr_2_-based PSCs, achieving an impressive 20.32% efficiency. Similarly, Elango’s group^107^ reached 28% efficiency through simulation-based optimization of thickness, defect density, bandgap, and electron affinity. Using the same simulation tool, another group reported ∼17% efficiency in all-inorganic CsSnBr_3_-based PSCs.^219^ Lead-free PSCs based on germanium show promise and good potential. Chen et al.^109^ found that CsGeI_3_ has good optoelectronic properties with an efficiency of 0.11%, which Zhao et al.^110^ improved to 4.92% using solvothermal synthesis. Another researcher reported that the substituted hazardous Pb^2+^ ions with monovalent (B^+^) and trivalent (B^3+^) cations, resulting in double perovskites (DPs) with the formula A_2_B^+^ B^3+^ X_6_, referred to as elpasolites.^111^ These have better stability but usually lower efficiency than lead-based PSCs. Yang et al.^112^ achieved 1.44% efficiency in Cs_2_AgBiBr_6_ DPs, while Zhang et al.^113^ improved it to 2.84% by using a N719 dye layer to enhance light absorption. Zhao et al.^114^ document hydrogenated Cs_2_AgBiBr_6_ exhibiting an efficiency of 6.37%. Recent DFT investigations conducted by Gardner et al.^341^ underscore Cs_2_TiI_6_ as a promising lead-free double perovskite, forecasting efficiencies of 22.70% for single-junction and 26.87% for tandem solar cells. These advancements indicate that green solvent engineering significantly used in efficiency, stability, and environmental safety, suggesting a sustainable future for perovskite solar cells.

#### 3.7.4 Green Solvent Engineering

Because of its ease of use and speed, solution processing and casting are widely used to create perovskite films. However, it commonly uses dangerous solvents, including *N*,*N*-dimethylformamide (DMF), chlorobenzene (CB), and *N*-methyl-2-pyrrolidone (NMP), which are harmful to human health and the environment both during production and disposal. For example, NMP increases energy use and environmental pollution, whereas DMF causes serious health problems. Researchers have looked into environmentally friendly solvents that create high-quality, compact films with a favorable PCE, such as ethanol and water. Triethyl phosphate (TEP), *n*-butyl acetate, and γ-valerolactone (GVL) are less harmful solvents generated from biomass that improve film quality and stabilize perovskite precursors. In addition to being more environmentally friendly, ionic liquids and custom solvents enhance solubility and recycling. Traditional antisolvents like toluene and chlorobenzene are dangerous and flammable, but using more eco-friendly substitutes like ethyl acetate, acetic acid, or polymer-based systems improves crystal formation, minimizes defects, and guarantees safer processing. Module efficiencies of 21% have been attained by research integrating green solvents for both precursors and antisolvents, hence removing exposure to dangerous chemicals. Techniques that do not use solvents, such as vapor deposition and melt processing, efficiently control layer thickness. However, these require expensive equipment and more energy. The sustainability of PSC production can be improved, and the environmental effect can be greatly decreased by combining green solvents with solvent recycling.^342–351^

#### 3.7.5 Commercialization Barriers

The cost of electrical devices depends on how long it lasts and how effective it is. Raw materials, production, labor, overhead, operational costs, and end-of-life practices like recycling are all included in the total cost. Expensive back-contact electrodes made of silver or gold are a major factor. For the sake of commercialization, the total cost of each device is not justified. As a result, less expensive alternatives like copper or carbon-based polymers are being researched. Environmental and economic problems arise from lead toxicity; recycling lead is crucial, but it is only profitable in a thriving recycling market.^354,355^ Cost-effectiveness is achieved by reusing fluorine-doped tin oxide (FTO) glass, a pricey substrate, without experiencing performance loss. Sofia et al. found that production costs can be significantly reduced by using less costly substitutes, such as barrier foils and conductive oxide-coated polymers, in place of more costly components.^356^ Reese et al. showed that combining low-cost multicrystalline silicon with perovskite in tandem cells can produce affordable, high-performance solar modules.^357^

##### 3.7.5.1 Lack of Industrial Standards

The absence of established procedures for testing stability and dependability continues to be a barrier to the commercialization of perovskite solar cells (PSCs), despite the fact that these cells have made significant advancements in power conversion efficiency (PCE). PSCs are examined using techniques that are not consistent, in contrast to silicon solar cells, which are required to comply with stringent testing standards established by the International Electrotechnical Commission (IEC). During the International Summit on Organic Photovoltaic Stability (ISOS), four different testing categories were introduced to address this issue. These testing categories are as follows: ISOS-D for dark storage, ISOS-L for light exposure, ISOS-T for thermal stress, and ISOS-O for outdoor testing. The purpose of these testing categories is to replicate real-world conditions in laboratories.^358,359^ On the other hand, a significant number of studies continue to rely on assessments that are either brief or nonstandardized, which makes it challenging to compare the outcomes of various research groups.

##### 3.7.5.2 Cost vs Silicon

PSC technology offers a more economical alternative to conventional photovoltaic systems such as crystalline silicon (c-Si) because of its low manufacturing costs, reduced material consumption, and lower production energy requirements. Large-scale production may be supported and prices can be further decreased with careful material selection. According to literature report, replacing pricey components with affordable alternatives such as barrier foils and transparent conductive oxide-coated plastic substrates can considerably cut total production expenses. However, volatility in raw material prices might undermine market stability. Perovskites with inexpensive multicrystalline silicon as the bottom cell can be combined to make the technology both commercially feasible and attractive to investors, according to International Electrotechnical Commission's (2016) analysis of perovskite–silicon tandem modules. According to these findings, the effective commercialization of PSC technology depends on lowering production costs without sacrificing performance.^360–362^

### 3.8 Policy and Sustainability

#### 3.8.1 Recycling Protocols and Pb Sequestration

Lead is the primary environmental risk associated with the materials used in hybrid perovskite solar cells (PSCs), which are frequently expensive and hazardous. Recycling these items decreases resource waste, production costs, and environmental impact. This section focuses on recovering and recycling lead to reduce its impact because it is the most hazardous component of discarded PSCs. As seen in Figure 12, a number of solar cell components are recyclable and reusable, while many others involve costly or dangerous ingredients. This enhances the general sustainability and worth of solar technologies in addition to lessening environmental harm.^363–365^ FTO glass accounts for 58% of PSC cost estimates, whereas metal and HTL electrodes account for 23% and 15% of total costs, respectively.^367,368^ Recycling reduces the risks associated with lead exposure. Manufacturing costs can be significantly decreased with this recycling process. Even with the development of lead-free perovskite technology, sustainable marketing of lead-containing perovskite solar cells depends on their safe disposal and recycling. Advanced encapsulating techniques, including hybrid polymer-inorganic barriers and ALD of Al_2_O_3_, greatly decrease lead leakage during use and disposal. Recycling techniques such as solvent dissolution, chemical precipitation, and selective ion extraction recover lead and recyclable precursors. Both lead-based and lead-free perovskite solar systems can be manufactured sustainably and with closed-loop material management when these environmental protections are incorporated into PSC design.

**Figure 12:**
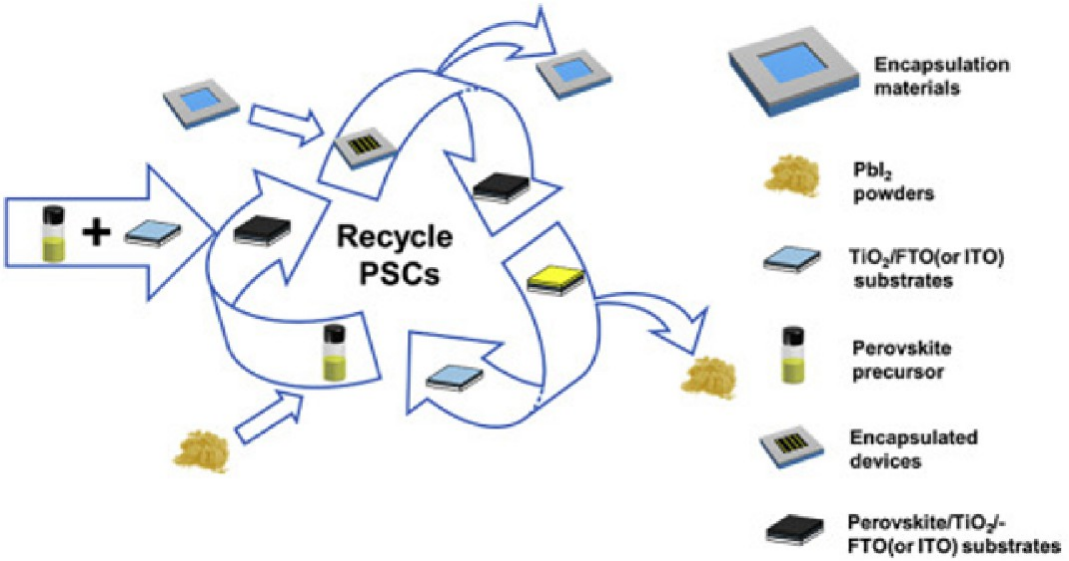
Schematic Recycling of PSCs, created by author.

#### 3.8.2 Government Incentives and Regulatory Frameworks

Government subsidies are essential for hybrid types of PSCs, by reducing financial obstacles for emerging technology. Countries such as India, through its Production-Linked Incentive scheme, and the European Union via Horizon Europe, finance research and development as well as the commercialization of advanced solar technologies, including perovskite-like silicon tandems. These initiatives reduce risks and promote private investment, industry acceptance, and pilot testing. Subsidies speed up PSC scaling, allowing them to compete with silicon photovoltaic technology, as Germany and the UK have shown. This assistance helps close the gap between commercial entry and laboratory success. Life-cycle analysis and ethical sourcing are related to sustainability. In 2023, NREL studies suggest lead management and carbon-neutral encapsulants could lower the environmental impact of silicon PSCs by 2030. Future research must focus on scalable manufacturing such as slot-die coating, roll-to-roll printing), cost reduction, and long-term reliability. Adopting standard tests (ISOS, IECs) ensures durability in real-world conditions. Overall, coordinated innovation in materials, device design, and policies, plus advances in recycling and encapsulation, plus government backing, will drive PSCs from the lab to large-scale use.^367^

## 4 Future Perspectives

New strategies of hybrid PSCs in material design, device engineering, and policy are enabling the development of highly stable, efficient, and sustainable photovoltaic technologies. This section highlights these progressions with an emphasis on the latest research outcomes and applications not only in wearing but also as self-healing perovskites.

### 4.1 Material Design

#### 4.1.1 Machine Learning (ML) and High-Throughput Screening

ABX_3_ perovskites to develop an effective ML model predicting thermodynamic stability. Zhao et al.^369^ reported four classification and four regression algorithms via 5-fold cross-validation. The Gradient Boosting Classification (GBC) model achieved the highest accuracy (0.872) and AUC (0.939) for identifying stable perovskites, while the extreme Gradient Boosting Regression (XGBR) excelled in predicting E_hull_ values with an R^2^ of 0.93 and Root Mean Square Error (RMSE) of 0.108 (Figure 13A). Combining both models enhanced prediction accuracy. Further analysis showed that perovskite composition strongly affects stability.

**Figure 13:**
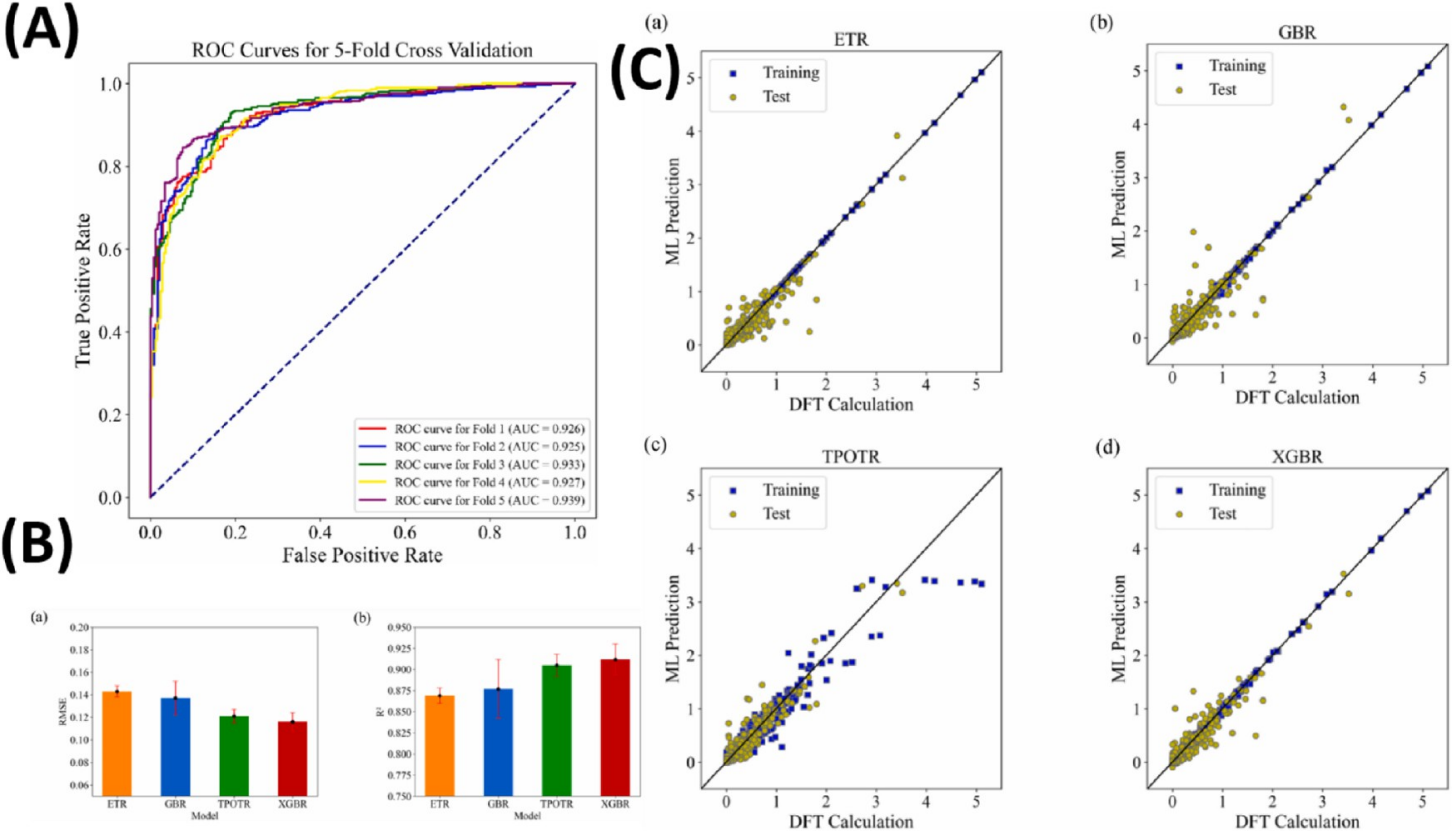
(A) The 5-fold cross-validation ROC curve for the GBC model. (B) Performance metrics derived from the average values of 5-fold cross-validation are compared with a- RMSE and b- R^2^ for four regression models. The 5-fold cross-validation standard deviation is shown by the red type I line. (C) shows the results of fitting the DFT value of E_hull_ to the expected value in the a- ETR, b- GBR, c- TPOTR, and d- XGBR models. Reproduced with permission, Copyright,[Bibr ref368] 2024.

As a result, the AUC was utilized to offer a thorough assessment of these four classification models’ performances. 5-fold cross-validation was used to get the AUC values for each GBC classifier, as shown in A. It is clear that the GBC classifier’s AUC accuracy, with an average value of 0.93, ranges between 0.925 and 0.939. Furthermore, Figure 13B­(a) shows the RMSE findings for the four regression models. The greatest accuracy determined using AUC values for the ETC, TPOTC, and XGBC models is 0.858, 0.931, and 0.897, respectively. The XGBR model appears to have far lower mistakes than the other three types. The comparison of R^2^ for the four regression models in Figure 13B­(b) makes it abundantly evident that XGBR obtains the greatest R^2^ value.

To evaluate the fitting performance, the real (DFT-calculated) and anticipated values of E_hull_ for the test data set were also shown. Figure 13C displays the ETR, GBR, TPOTR, and XGBR models’ fitting quality. Among them, the XGBR model’s projected E_hull_ values closely match the test data set’s actual values, indicating the model’s improved predictive performance for estimating the E_hull_ of halide perovskites with ABX^3^ structure in regression models.^368^

#### 4.1.2 Bio-Inspired and Self-Healing Perovskites

The concept of self-healing materials inspired by biological systems is gaining attention in PSC design. Recent studies explore incorporating dynamic bonds and supramolecular interactions within the perovskite lattice to allow defect repair. For instance, in an intriguing study, Asare et al.^370^ showed how crucial it is to use polyethylene glycol (PEG) as a low-cost, long-chain, and hygroscopic polymer scaffold when creating MAPbI_3_ perovskite in order to stop spin-coated films and n-i-p cells from degrading. The one-step spin-coating process was used to incorporate PEG into the perovskite precursor solution. More significantly, because of PEG’s potent hygroscopicity, the insulating PEG scaffold structure may stabilize the PSCs in humid environments. Here, three sets of unsealed devices were made, one with PEG (at two concentrations) and the other without. The PCE evolution of the three sets of devices in the dark, in a very humid environment (relative humidity 70%), without any sealing, is displayed as a function of time in Figure 14(A). The PPSCs maintained 65% of their PCE after 300 h of age, in contrast to the pristine PSCs’ rapid PCE loss from 12 to 0.5% after 50 h. Figure 14(B) illustrates how these devices’ increased stability and capacity for self-healing in the presence of moisture were proven. The irreversible degradation of the PEG-free perovskite film to PbI_2_ is undeniable. On the other hand, the PEG-MAPbI_3_ film became black as soon as the source of humidity was removed, despite first appearing yellow after some time of exposure to water vapor.

**Figure 14:**
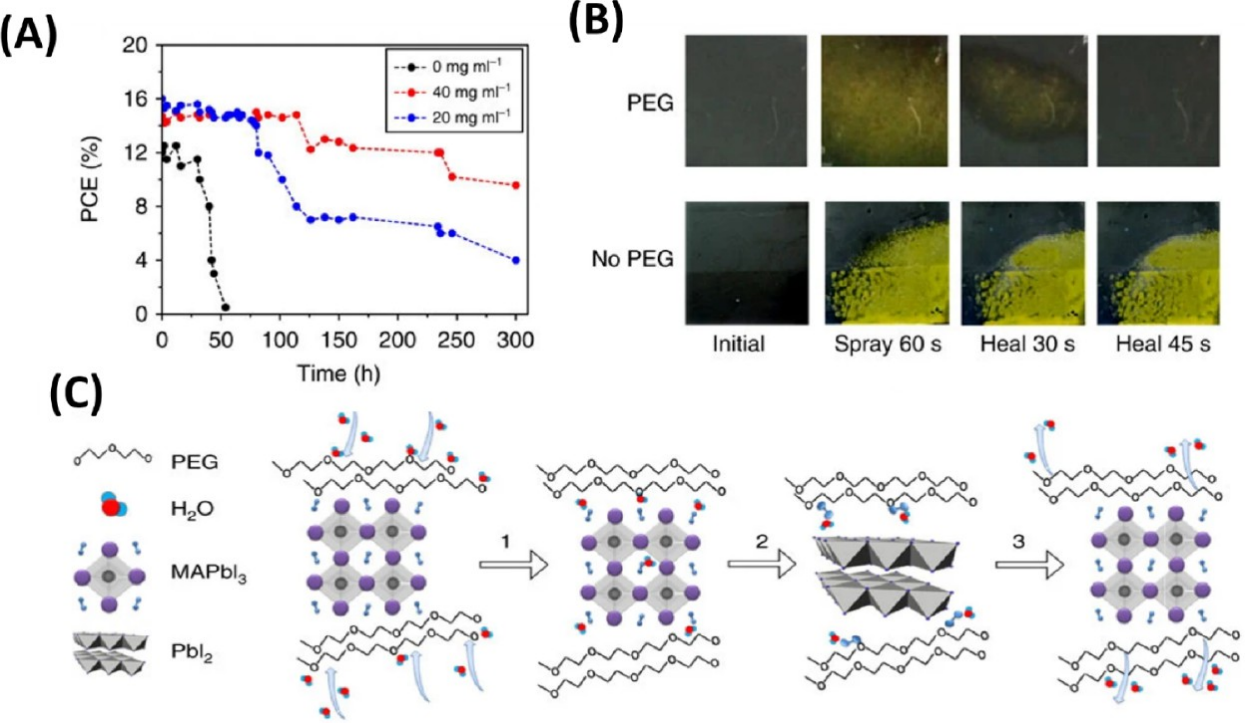
(A) PSCs with 20 or 40 mg mL^–1^ PEG and without PEG scaffold subjected to a high relative humidity (70%), dark environment without any sealing, showing the development of PCE over time. Images of perovskite films with and without PEG demonstrate the progression of color change during 60 s of water spraying and 45 s of ambient air exposure. (B) A schematic illustration of the suggested processes behind PSCs’ self-healing capabilities. (C) PEG–MAPbI_3_ film’s self-healing process (reversible degradation). Reproduced with permission, Copyright,[Bibr ref369] 2016.

Significantly, the PCE restoration to its initial value is also displayed by the current density–voltage curves of PSCs, indicating that the PEG–MAPbI_3_ films and the associated devices both self-healed, restoring the PCE following the water vapor removal. The PEG polymer’s high hygroscopicity and strong contact with MAI, as shown by nuclear magnetic resonance (NMR) tests, were credited with the self-healing ability. A significant interaction between MA^+^ and O of the PEG chain was shown by the 1H-NMR spectra, which displayed a chemical shift and a splitting of the signal associated with the proton of the methylene group connected to the oxygen of the PEG, −[O–CH_2_–CH_2_]_n_–. The PEG–MAPbI_3_ film’s self-healing process (reversible degradation) is depicted in Figure 14C.^369^

### 4.2 Device Engineering

#### 4.2.1 Ultra-Stable Encapsulation – ALD Coatings

Encapsulation plays a pivotal role in protecting PSCs from environmental degradation. Atomic layer deposition (ALD) of Al_2_O_3_ has emerged as a superior method for forming ultrathin, conformal, and pinhole-free barrier layers. Atomic layer deposition (ALD) of aluminum oxide (Al_2_O_3_) and other appropriate metal oxides, such as zirconium oxide (ZrO_2_), as a perovskite surface passivation approach, Asare et al. has led to recent developments in perovskite solar cells (PSCs). With just a few nanometers of thickness, ALD has shown great promise for improving photovoltaic (PV) performance and the long-term light, thermal, humidity, and ultraviolet (UV) stability of PSCs by addressing surface defects that increase charge extraction and reduce environmental degradation.^370^ ALD coatings also serve as moisture barriers in flexible devices without sacrificing mechanical integrity.

#### 4.2.2 Flexible PSCs – Wearable and BIPV Applications

The integration of PSCs into wearable electronics and building-integrated photovoltaics (BIPV) demands mechanically robust and efficient designs. While flexible PSCs have been developed for wearable and portable electronics due to their conformability, for BIPV applications rigid PSCs are generally sufficient and more practical. Nonetheless, flexible PSCs could offer potential advantages in curved or unconventional architectural surfaces, although their large-scale implementation in buildings is currently limited. Recent progress includes the development of ultrathin flexible substrates (e.g., PEN, PET) combined with low-temperature processed perovskites.

### 4.3 Wearable Applications

Perovskite solar cells (PSCs) are flexible, thin, and lightweight. Consequently, PSCs offer a number of exciting opportunities for customers and wearable technology with their excellent energy conversion efficiency. PSCs are ideal for driving small, portable devices like smartphones and smartwatches. Moreover, due to the requirement of wearable technology for dependable power source under low-light conditions, the excellent low-light performance makes them an appropriate choice for this application as well. A portable self-sustaining pressure sensor based on the combination of a thin-film organic solar cell and a piezo-transmittance microporous elastomer was disclosed by Choi et al. In this, a thin-film organic solar cell (OSC) and piezo-transmittance microporous elastomer (PTME) are integrated to create a wearable, self-powered pressure sensor [371]. Figure 15 The schematic design of the piezo-transmittance-based self-powered pressure sensor is illustrated in Figure 15(A). The suggested self-powered pressure sensor uses natural light as its power source, independent of its intensity, and is capable of continually and steadily monitoring static pressure, in contrast to sensors based on other methods like piezoelectricity or triboelectricity. The PTME responds to applied pressure by gradually closing micropores through compression, which alters light transmittance. Because of the PTME’s special optical properties, the OSC may produce different electrical currents depending on the pressure. High performance is demonstrated by the suggested self-powered pressure sensor, which has a linearity of R^2^ = 0.995, a sensitivity of 0.101/kPa, and is quick.

**Figure 15:**
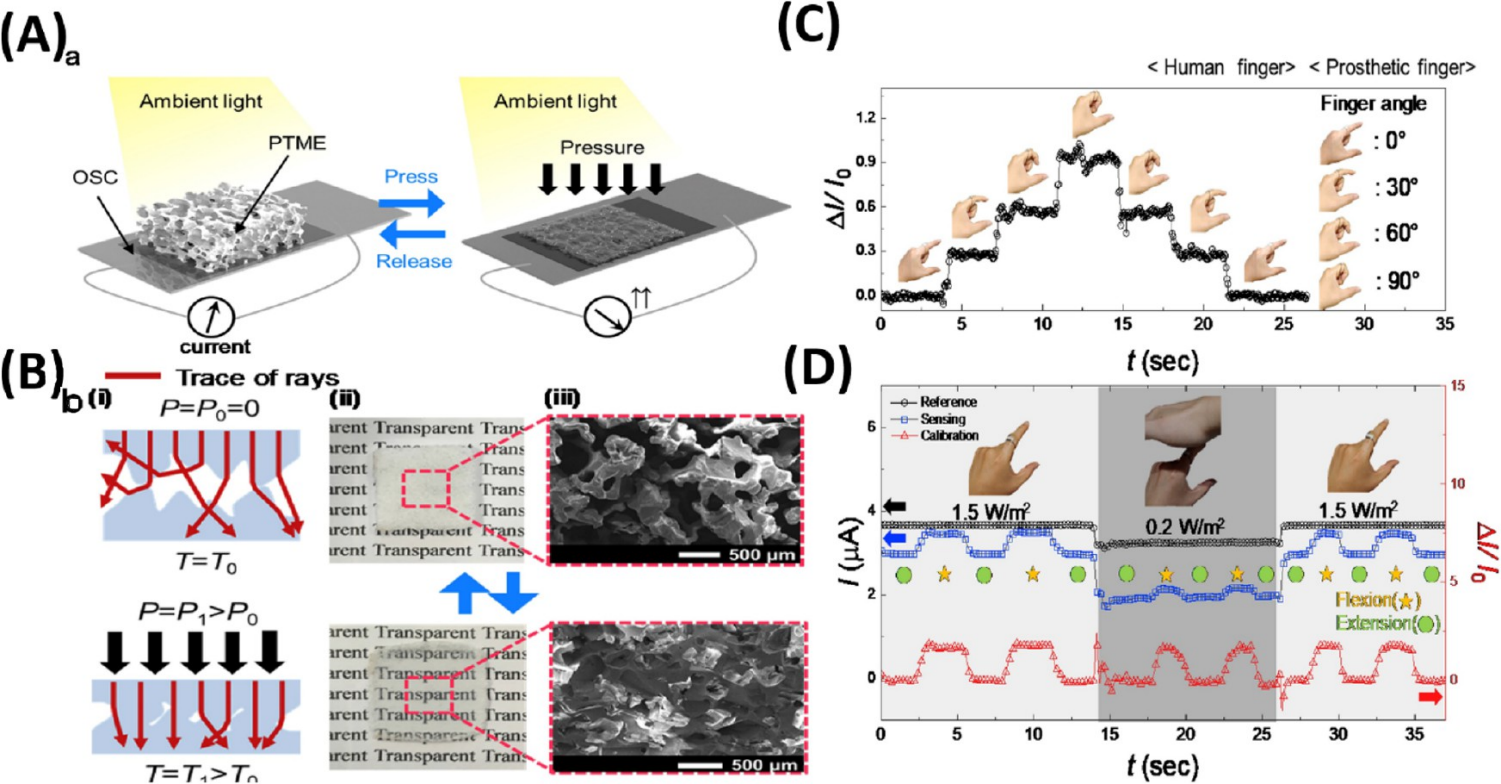
(A) Schematic depiction of self-sustaining pressure sensor based on piezo-transmittance PTSPS. (B) The PTME’s optical characteristics under compression. (i) Light scattering under the uncompressed state (*P* = P0 = 0) results in low light transmission through the PTME (*T* = T0), but under the compressed state (*P* = P1 > P0), light transmission through the PTME is greater (*T* = T1 > T0). (ii) Images showing the PTME’s transparency change both before and after compression (top and bottom, respectively). (iii) SEM pictures demonstrating the PTME’s pore-closing activity both before and after compression (top and bottom, respectively). (C) The sensor’s reaction over the 0°–90° finger angle. (D) The calibrated response using repetitive flexion/extension motions of the human finger under varying light intensity, as well as the sensor responses of the detecting OSC and reference OSC. As the light intensity fell, so did the current produced by the reference OSC and sensing OSC. However, as the red line with a triangle marker illustrates, the calibrated relative change of current has a similar trend independent of light intensity. Reuse with permission, Copyright,[Bibr ref371] 2020.

A schematic of the PTSPS is shown in Figure 15A. This sensor is made of two main parts: the PTME, which allows light to pass through, and the organic solar cell (OSC), which generates electricity from light. The soft, flexible, and translucent PTME film was made using a sugar cube mold. Under normal circumstances, microscopic pores of the film scatter light. These pores partially close in response to external pressure, which lessens light dispersion and increases light transmission. Consequently, more light reaches the organic solar cell, increasing the generation of photocurrent. This is how the pressure sensor operates: more pressure results in higher current output and more light transmission. The active layer of the organic solar cell (OSC) is PTB7-Th, which has a thin, flexible structure that makes it ideal for wearable electronics applications and is extremely sensitive to weak light intensities (around 1 W/m^2^). In Figure 15 B­(i), represents how light passes differently through the unpressed and pressed PTME: At rest (P = P_0_ = 0), the light is scattered by the pores, and little reaches the OSC. Under pressure (P = P_1_ > P_0_), pores close, scattering is reduced, more light reaches the OSC, higher transmittance, T_1_ > T_0_, and more current is generated. When pressure is released, the PTME returns to its original shape thanks to its elastic nature. Figure 15B (ii) and Figure 15B­(iii) show how the PTME changes between unpressed and pressed states, captured using transparent acrylic plates and SEM images. Initially, the micropores are about 400 ± 140 μm in size, and the PTME has a porosity of 72.8 ± 0.5%.

To verify their effectiveness, researchers also conduct various experiments. Some of them reported that the PDMS film remains in its initial location when the finger is not flexed. PDMS films can be flexed to extend horizontally, compressing the PTME vertically and altering the PTME’s transmittance. Thus, while the light intensity is constant, the finger’s flexing movement simply modifies the detecting OSC’s response. The prosthetic finger may be adjusted to match the human finger angle by evaluating the finger angle based on the reaction (Figure 15C). It should be mentioned, nevertheless, that the illumination conditions—such as varying levels of interior lighting or solar irradiation—can cause the incoming light’s intensity to fluctuate continually. To counteract this impact, we have used a pair of reference OSC and sensing OSC. The current produced by the reference OSC and the sensing OSC, respectively, with repetitive flexion/extension motions of the human finger under varying light intensities is shown in Figure 15(D) by the black curve with circle markers and the blue curve with square markers. As the light intensity dropped, so did the current produce by the sensing OSC and the reference OSC.^371,372^

### 4.4 BIPV Applications

Buildings use nearly one-third (about 40%) of global energy, so improving their energy efficiency is a major challenge today. One solution is using solar energy systems that are built into buildings, which can help produce cheaper electricity using photovoltaic (PV) technology.^373,374^ Building-integrated photovoltaics (BIPVs) refer to materials that serve two purposes—both as building materials and as energy generators. BIPVs have gained a lot of attention as a clean energy option for buildings in crowded areas where traditional ground-mounted PV systems cannot be used.^375–377^ BIPVs can provide around 22% or more of global electricity demand by 2030.^375,378–381^ BIPV design requires some factors, such as building orientation, location, cost-efficiency, energy requirements, regulatory standards, and electrical load demands. Semitransparent perovskite solar modules (PSMs) are gaining traction due to their flexibility and appealing visual qualities, making them well-suited for BIPV applications.^382–384^ Sunlight entering any building can be controlled using solar glazing. This glazing system combines glass and PVs to create power-generating windows.^386,387^ PSMs can be added to solar-glazing windows, offering both flexibility and partial transparency.^387,388^ Lightweight PSMs can also replace ceramic tiles or gravel on rooftops, giving practical and attractive roofing options for BIPVs.^389–392^ While c-Si modules are still on the market, they do not offer the best balance between cost and performance.

PSCs have been used in smart windows that control light entry and in solar panels that generate electricity. To create nanostructured perovskites for ST PSC manufacturing, Moon and colleagues^390^ created parallel aluminum oxide nanopillars as a scaffold layer (Figure 16A-B). The thickness of the perovskite layer regulated the device’s transmittance (Figure 16A-B). The optimized device has an AVT of 33.4% and a PCE of 9.6%. The creation of a unique design for perovskite growth should receive greater attention since structural control over perovskite layers is a potential method for ST PSCs. For the creation of ST PSCs, several tried-and-true techniques for precisely regulating the perovskite development, such as etching and efficient deposition, ought to be investigated^392^ . With the use of outdoor test findings, Kwon et al. (2016)^391^ illustrate the effective structures and procedures for dependable perovskite solar modules in BIPV facade applications, which paves the way for technological validation for a fully integrated solution (Figure 16C). The scientists presented a 35 × 35 cm^2^ perovskite-based module design that is economical, highly efficient (>17%), and has been reliable for more than 20 years.

**Figure 16:**
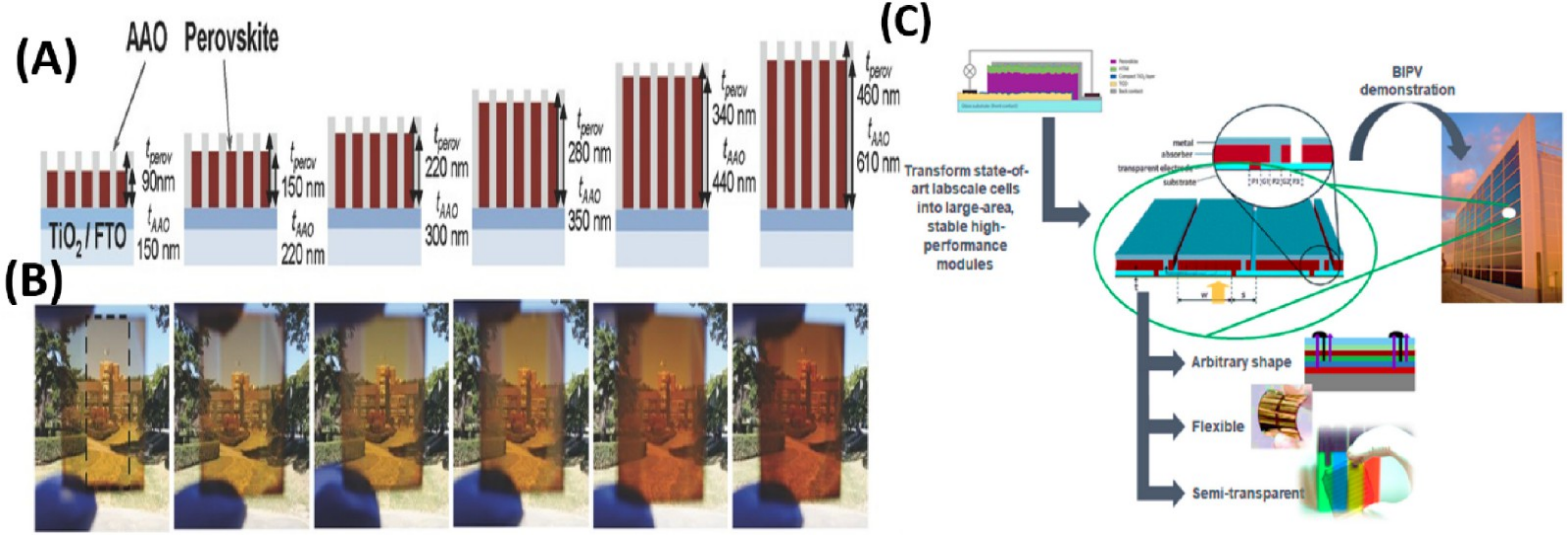
(A, B) Schematic representation of various thicknesses of spin-infiltrated perovskite layers in anodic aluminum oxide (AAO) templates; matching perovskite films are also shown. Wiley-VCH, 2016. Reproduced with permission Copyright,[Bibr ref390] 2019. (C) The effective structures and procedures for dependable PSC modules for BIPV application are depicted in this. Reproduced with permission, Copyright,[Bibr ref391] 2016.

## 5 Conclusion

Hybrid PSCs have rapidly advanced to become a leading photovoltaic technology, driven by exceptional optoelectronic properties and power conversion efficiencies exceeding 26%. PSCs are promising materials due to their high efficiency, improved stability, scalable manufacturing, and sustainability. The efficiency of PSCs is enhanced by defect passivation, composition, and advanced charge carrier management. Stability of hybrid PSCs improvements stems from encapsulation, additives, and compositional adjustments addressing moisture, heat, and mitigation. Scalable techniques for large-scale development of PSCs, possibly done by techniques like slot-die and blade coating, ensure uniform, reproducible large-area production. Sustainability of hybrid PSCs focuses on lead management, recycling, and green solvents to mitigate environmental impact. Integrating these aspects through smart and good PSC material design and robust fabrication paves the way for commercially viable, durable, and eco-friendly PSCs, positioning them as key future solar technologies.

## Data Availability

No data were used for the research described in the article.
